# Supplementary material for: Impaired mitochondrial metabolism is a critical cancer vulnerability for MYC inhibitors
Source: Sci Adv. 2025 Jul 16;11(29):eadw5228. doi: 10.1126/sciadv.adw5228 (PMC12266110; doi:10.1126/sciadv.adw5228)
Supplement: 20250716-1 [file sciadv.adw5228.v1.pdf]

CANCER

Impaired mitochondrial metabolism is a critical cancer vulnerability for MYC inhibitors

William Yang[1,2], Qianyu Guo[1,2], Songhua Quan[1,2], Zachary R. Chalmers[1,2], J. Brandon Parker[3], Mihai Truica[1,2], Mary F. Dufficy[1,2], Megan M. Kerber[1,2], Karthik Vasan[2,4], Dikshat G. Gupta[1,2], Adam W. T. Steffeck[1,2], Hao Pan[1,2], Mohammed Siddiqui[1,2], H. Tran Pham[5], Gary E. Schiltz[6], Debabrata Chakravarti[2,3,7], Navdeep S. Chandel[2,4], Sarki A. Abdulkadir[1,2]*

Copyright © 2025 The Authors, some rights reserved; exclusive licensee American Association for the Advancement of Science. No claim to original U.S. Government Works. Distributed under a Creative Commons Attribution License 4.0 (CC BY).

MYC is a key driver in many aggressive and therapy-resistant cancers. We have developed and characterized a small-molecule MYC inhibitor named MYCi975. To uncover combination strategies for MYC inhibitors, we conducted a genome-wide CRISPR screen using MYCi975. This screen revealed a notable synthetic lethality when MYC inhibition was paired with disruption of mitochondrial complex I components, but not other complexes. Mechanistically, MYC inhibition reduced oxidative phosphorylation and glycolysis, triggering a compensatory up-regulation of complex I genes. Consequently, genetic or pharmacological targeting of complex I sensitized tumors to MYCi975 treatment, leading to increased purine catabolism and infiltration of CD8$^+$ T cells and macrophages into tumors. Additionally, a wide range of tumor cells with lower complex I expression showed increased MYC dependency. These results indicate that metabolic adaptation to MYC inhibition exposes a targetable weakness at complex I and provide a rational strategy for combination therapy with emerging MYC inhibitors.

INTRODUCTION

MYC deregulation is a hallmark of aggressive cancers and drives tumor growth and therapy resistance across multiple cancer types (*1*, *2*). Although MYC has long been considered an "undruggable" target, recent breakthroughs have yielded several promising MYC inhibitors that are advancing in clinical development (*3*–*6*). For example, OmoMYC, a dominant-negative MYC inhibitor peptide, has demonstrated encouraging clinical activity, supporting the concept of direct MYC targeting as a viable therapeutic strategy (*7*, *8*). As these emerging agents progress through clinical development, identifying rational combination approaches to maximize their therapeutic potential represents a critical opportunity to improve patient outcomes.

MYC orchestrates a vast array of cellular processes, including cell cycle progression, apoptosis, DNA replication, protein synthesis, and metabolism (*9*–*11*). This broad control over cellular functions has made it challenging to predict which pathways, when targeted, might synergize with MYC inhibition to enhance the therapeutic efficacy. Among MYC's many roles, its regulation of cellular metabolism is particularly profound (*12*, *13*). Although MYC activation of glycolysis in cancer cells is well documented, its control over mitochondrial oxidative phosphorylation (OXPHOS) is poorly understood (*12*, *13*). MYC directly regulates nuclear-encoded mitochondrial genes and drives mitochondrial biogenesis, enabling cancer cells to maintain a high OXPHOS capacity despite elevated levels of glycolysis (*14*, *15*). This dual metabolic control allows MYC-driven tumors to flexibly use both energy-generating pathways to support aggressive growth and survival (*12*). Moreover, genetic studies in *Myc*$^{-/-}$ fibroblasts have demonstrated that MYC is essential for maintaining normal mitochondrial structure and function, further underscoring its pivotal role in cellular bioenergetics (*16*).

Although multiple functional genomic screens have been conducted to identify vulnerabilities in *MYC*-overexpressing cells, systematic identification of pathways that synergize with MYC inhibition remained unexplored (*17*–*19*). This represents a critical knowledge gap, particularly in light of the advancement of MYC inhibitors in clinical development (*3*, *8*). To systematically identify the pathways that could enhance MYC inhibitor efficacy, we performed an unbiased genome-wide CRISPR screen in MYC-dependent cancer cells treated with the small-molecule MYC inhibitor MYCi975 (*4*, *19*–*21*). Our study revealed an unexpected connection between MYC inhibition and cellular/mitochondrial metabolism, identifying mitochondrial function as a key determinant of response to MYC inhibitors. We demonstrated that this vulnerability can be therapeutically exploited using clinically approved metabolic modulators such as metformin, establishing a paradigm for enhancing the efficacy of MYC-targeted therapies (*22*–*24*). This strategy, which combines emerging MYC inhibitors with well-characterized metabolic drugs, provides an immediate translatable approach to improve treatment outcomes in patients with MYC-driven cancers.

RESULTS

Genome-wide CRISPR screen identifies mitochondrial function as a vulnerability to MYCi975

Although several MYC inhibitors are entering clinical development, strategies to enhance their efficacy and expand their therapeutic window remain undefined (*3*, *8*, *25*, *26*). MYC regulates numerous cellular processes, including metabolism, cell cycle, and apoptosis; however, systematic identification of pathways that could synergize with MYC inhibition has not been thoroughly explored (*13*). To address this unmet need, we conducted a genome-wide CRISPR knockout (KO) screen in two biological replicates using murine MycCaP

[1]Department of Urology, Northwestern University Feinberg School of Medicine, Chicago, IL, USA. [2]The Robert H. Lurie Comprehensive Cancer Center, Northwestern University Feinberg School of Medicine, Chicago, IL, USA. [3]Division of Reproductive Science in Medicine, Department of Obstetrics and Gynecology, Northwestern University Feinberg School of Medicine, Chicago, IL, USA. [4]Division of Pulmonary and Critical Care Medicine, Department of Medicine, Northwestern University Feinberg School of Medicine, Chicago, IL, USA. [5]Division of Internal Medicine, Weiss Memorial Hospital, Chicago, IL, USA. [6]Department of Chemistry, Northwestern University, Evanston, IL, USA. [7]Department of Pharmacology, Northwestern University Feinberg School of Medicine, Chicago, IL, USA.
*Corresponding author. Email: sarki.abdulkadir@northwestern.edu

prostate cancer cells and the well-characterized small-molecule MYC inhibitor MYCi975 (Fig. 1A). We selected MycCaP because it is a robust MYC-driven cancer model that can be transplanted into syngeneic immunocompetent mice, facilitating both in vitro and in vivo analyses. We used the mouse Toronto KnockOut (mTKO) CRISPR library, targeting 19,463 mouse genes with 94,528 single guide RNAs (sgRNAs; five sgRNAs per gene) (*27*).

We determined the lethal dose that kills 20% of cells (LD_{20} dose) for MYCi975 in MycCaP cells to be 3.5 μM. The target of 10 cell-doublings for the screen was consistently achieved within a 9-day treatment period across biological replicates (fig. S1, A and B). The robust performance of our screen was validated by the identification of the expected essential genes, including core ribosomal components (*Rpl10* and *Rpl11*), RNA processing factors (*Pol3h*), and cell cycle regulators (*Cdk13*) (fig. S1C) (*28*, *29*).

Analysis of sequencing data revealed a clear separation between genes whose deletion enhanced MYCi975 potency ("synergistic hits") versus those that impaired drug efficacy, with synergistic hits displaying strongly negative NormZ scores (Fig. 1B and table S1) (*29*). Gene Ontology (GO) analysis of these synergistic hits revealed a notable enrichment for mitochondrial processes, with the mitochondrial respiratory chain complex assembly emerging as the most significantly enriched pathway (Fig. 1C). Most notably, this enrichment showed pronounced selectivity for components of mitochondrial complex I, in contrast to other respiratory chain complexes where synthetic lethality was minimal (0 to 12.5% of components). Complex I emerged as uniquely vulnerable, with 43.9% (29 of 66) of its components identified as synthetic lethal with MYCi975 in our screen (Fig. 1D, fig. S1D, and table S2). Although the screen identified additional mitochondrial vulnerabilities, including complex IV assembly factors and reactive oxygen species (ROS) regulators (Fig. 1B), the selective enrichment for complex I components suggests that it plays a unique role in mediating MYC inhibitor sensitivity.

Consistent with this view, transcriptional profiling of P493 and PC3 cells showed that MYCi975-mediated inhibition closely mirrored gene expression changes observed with genetic depletion of MYC (fig. S1, E and F, and table S2). When we examined a subset of genes according to different clusters (fig. S1, E and F), MYCi975 broadly repressed MYC-driven oncogenic programs, particularly those governing uncontrolled growth and replication (clusters 1 and 2), while partially sparing processes, such as ribosome biogenesis and mitochondrial metabolism (cluster 3). Thus, MYCi-treated cells were susceptible to further repression of cluster 3 gene function. Together, these data led us to postulate that cells become specifically vulnerable to the disruption of mitochondrial complex I when MYC is inhibited.

Loss of mitochondrial complex I components sensitizes cells to MYC inhibition

To validate the synthetic lethal interaction observed between mitochondrial metabolism and MYC inhibition, we focused on two key hits from our screen using the MycCaP castration-resistant prostate cancer cell line: *Ndufa3*, a core subunit of complex I (*30*), and *Sod2*, a mitochondrial superoxide dismutase that protects against oxidative stress (*31*). These genes represent distinct aspects of mitochondrial function: electron transport chain activity and ROS detoxification, respectively. As expected, MYC knockdown decreased cell viability; however, the combined depletion of MYC and *Ndufa3* using small interfering RNA (siRNA) and CRISPR-mediated KO led to a significant loss of MycCaP cell viability compared to individual knockdown (Fig. 2, A and B). While KO of either *Ndufa3* or *Sod2* alone was well tolerated, both sensitized the cells to MYC inhibition using two independent sgRNAs per gene (Fig. 2, C and D), confirming the synthetic lethality observed in our screens.

To determine whether this synthetic lethality required functional complex I disruption, we leveraged the unique properties of yeast NADH [reduced form of nicotinamide adenine dinucleotide (oxidized form)] dehydrogenase (NDI1). NDI1 is a single-subunit enzyme that can functionally replace the entire mammalian complex I in the electron transport chain, thereby providing electron transfer from NADH to ubiquinone (*32*, *33*). The expression of NDI1 specifically rescued the synthetic lethality of Ndufa3 loss and MYC inhibition, but not that of Sod2 (Fig. 2, E and F). This selectivity is expected because NDI1 can only restore complex I function, not general mitochondrial function, while Sod2 loss affects a distinct mitochondrial process of ROS management. Furthermore, NDI1 was able to rescue growth inhibition of *Ndufa3* KO cells treated with OmoMYC, a clinical-stage MYC inhibitor, confirming that the mechanism is broadly applicable to distinct MYC-targeting agents (fig. S2A). Together, our results indicate that compromised electron transport through complex I, rather than general mitochondrial dysfunction, drives synthetic lethality with MYC inhibition in Ndufa3-deficient cells.

To explore whether the synthetic lethality with Sod2 loss similarly depends on mitochondrial ROS, we treated cells with MitoTEMPO (5 μM), a mitochondria-targeted superoxide scavenger (*34*). While MitoTEMPO had no effect on control cells, it significantly rescued viability in *Sod2*-KO cells treated with MYCi975 (fig. S2B). This selective rescue confirms that excessive mitochondrial ROS specifically drives synthetic lethality in cells with compromised antioxidant capacity, representing a distinct but parallel vulnerability to complex I dysfunction.

To further explore the relationship between complex I function and MYC dependency, we analyzed the DepMap AVANA dataset, stratifying cell lines based on complex I gene expression (*35*). We focused on the extreme ends of the complex I gene expression spectrum (bottom and top 10th percentiles) to capture cell lines with distinctly different complex I activity levels while maintaining sufficient sample sizes for robust statistical analysis. We found that cell lines with low complex I expression exhibited markedly higher MYC dependency than those with high complex I expression (Fig. 2G and table S3). A volcano plot of the differential dependencies between complex I–low and complex I–high cell lines revealed *MYC* as one of the most pronounced dependencies in complex I–low cells (fig. S2C and table S3), with a negative delta dependency score indicating stronger reliance on MYC in these cells (Fig. 2G and fig. S2C).

To determine whether these observations can be extended to human tumors, we analyzed The Cancer Genome Atlas (TCGA) data. We first examined correlations between MYC protein levels and complex I gene expression across prostate adenocarcinoma (PRAD) samples. Most complex I subunits showed positive correlations with MYC protein levels, with core components *NDUFS7*, *NDUFV1*, and *NDUFS6* exhibiting the strongest associations (fig. S2D). When we stratified 493 patients with PRAD on the basis of complex I expression, those with lower expression showed significantly better progression-free survival compared to patients with higher expression (log-rank $P = 3.235 \times 10^{-3}$; fig. S2E). These clinical data suggest that increased complex I expression not only correlates with MYC levels but also predicts worse patient outcomes, providing a strong rationale for targeting this axis therapeutically.

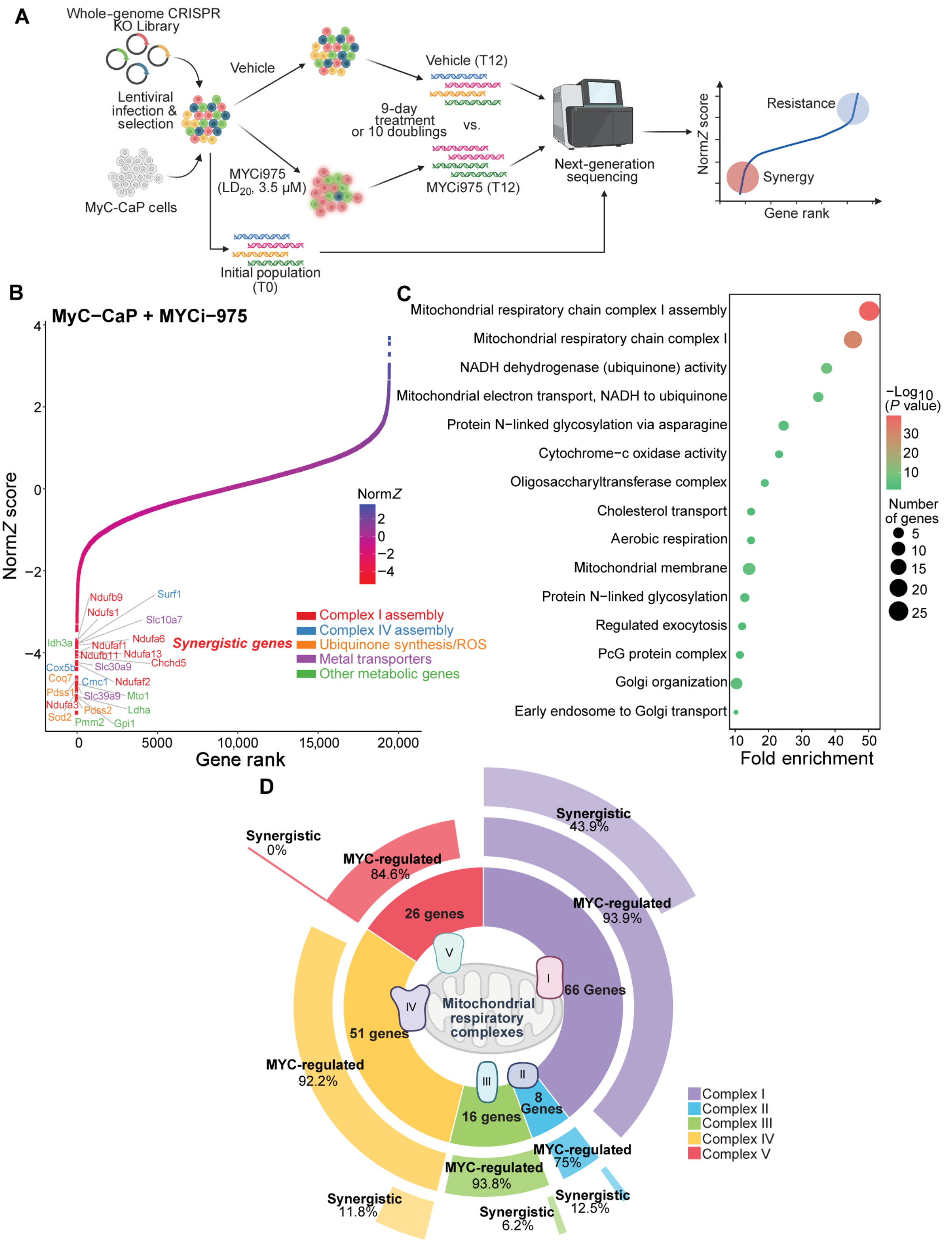

Fig. 1. Genome-wide CRISPR screen identifies mitochondrial complex I as synthetic lethal with MYC inhibition. (**A**) Experimental design for genome-wide CRISPR screen in MycCaP cells. Cells were infected with the mouse Toronto KnockOut (mTKO) CRISPR library, selected, and treated with either vehicle or MYCi975 at LD20 (3.5 μM) for 9 days or 10 population doublings before next-generation sequencing. Created in BioRender. W. Yang (2025), https://BioRender.com/6x6pegu. (**B**) Norm*Z* scores for all screened genes plotted against gene rank. Genes that synergize with MYCi975 (red) exhibit strongly negative Norm*Z* values. Key metabolic hits are color coded by function as shown. (**C**) Gene Ontology (GO) analysis of synergistic hits reveals significant enrichment of mitochondrial pathways. Point size corresponds to gene number and color intensity reflects statistical significance [$-\log_{10}$(*P* value)]. (**D**) Donut chart showing the proportion of MYC-regulated and synergistic components within each mitochondrial respiratory chain complex (I to V). Complex I exhibits the highest fraction of synthetic-lethal hits (43.9%), compared to ≤12.5% in other complexes.

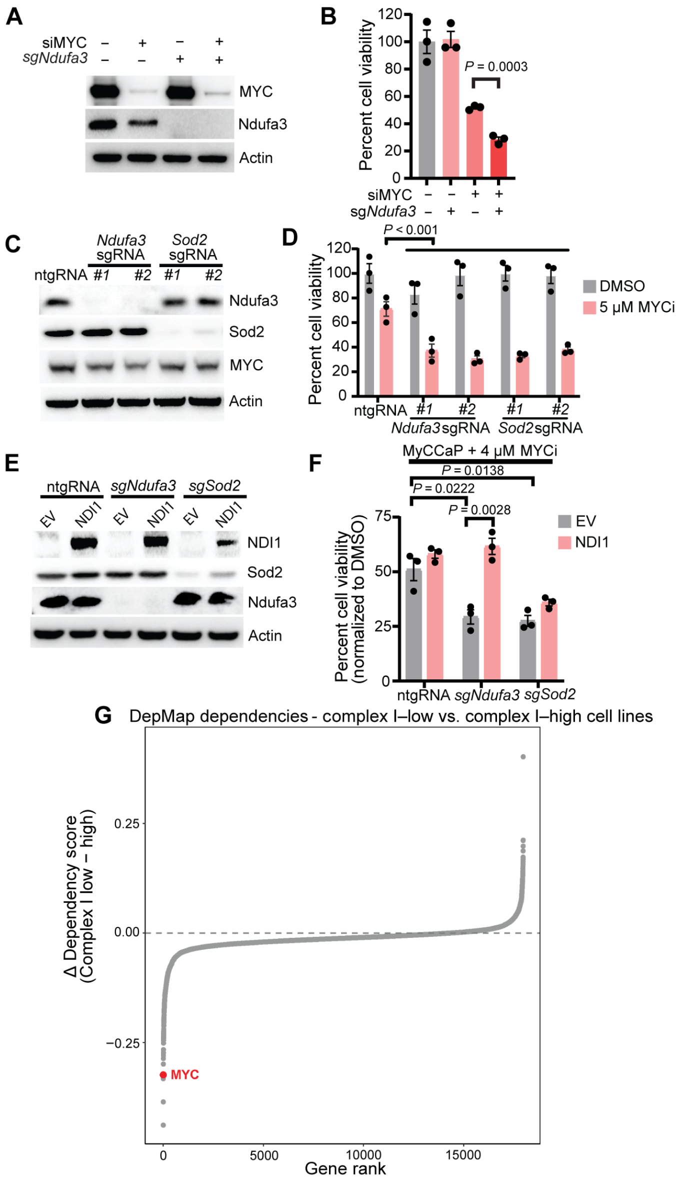

Fig. 2. Complex I disruption sensitizes cells to MYC inhibition. (**A**) Western blot confirming small interfering RNA (siRNA)–mediated MYC knockdown and sgRNA-mediated Ndufa3 KO in MycCaP cells. Actin is a loading control. (**B**) Cell viability (manual counting) in MycCaP cells subjected to indicated siRNAs/sgRNAs for 3 days. Data are means ± SEM ($n = 3$); unpaired two-tailed Student's t test. (**C**) Western blot validation of Ndufa3 or Sod2 KO in MycCaP cells using two independent sgRNAs per gene. Actin is a loading control. (**D**) Cell viability of MycCaP cells expressing control or Ndufa3/Sod2 sgRNAs treated with dimethyl sulfoxide (DMSO) or 5 μM MYCi975. Data are means ± SEM ($n = 3$); one-way analysis of variance (ANOVA), Tukey's test. (**E** and **F**) Expression of yeast NADH [reduced form of nicotinamide adenine dinucleotide (oxidized form)] dehydrogenase (NDI1) rescues synthetic lethality between Ndufa3 loss and MYC inhibition but not Sod2 loss. Immunoblot validation (E) and quantification of cell viability (F) in cells treated with 4 μM MYCi975. Data are means ± SEM ($n = 3$); one-way ANOVA, Tukey's test. (**G**) Analysis of DepMap AVANA dataset showing differential gene dependencies between complex I–low and complex I–high cell lines. MYC exhibits enhanced dependency in complex I–low cells (red dot). Data visualized by authors; original CRISPR-dependency scores from DepMap AVANA (*35*).

Last, among our top hits, *Ldha* stood out as a nonmitochondrial gene showing strong synthetic lethality with MYCi975 (Fig. 1B). To understand why *Ldha* loss sensitizes cells to MYC inhibition, we tested inhibitors of three pyruvate metabolic pathways in combination with MYCi975 (fig. S2F). Lactate dehydrogenase A (LDHA) inhibition produced the strongest synthetic lethality (viability ~10%, $P = 0.0002$), followed by Pyruvate Dehydrogenase (PDH) inhibition (viability of ~35%), while pyruvate carboxylase inhibition showed dampened synergy (viability of ~45%). This suggests that redirecting pyruvate toward mitochondrial metabolism creates metabolic vulnerability in MYC-inhibited cells. Together, these results establish a synthetic lethal interaction between mitochondrial complex I dysfunction and MYC inhibition, suggesting that cells with compromised complex I function become critically dependent on MYC for survival.

MYC inhibition disrupts mitochondrial structure and bioenergetics

To understand the mechanism underlying the synthetic lethality between MYC inhibition and mitochondrial dysfunction, we first examined whether MYCi975 directly inhibited mitochondrial complex I function using isolated mitochondria. Unlike rotenone, a complex I toxin that directly binds and reduces complex I activity by ~80%, MYCi975 did not directly inhibit complex I enzymatic activity, even at concentrations up to 10 μM (Fig. 3A) (*15*, *36*). However, transmission electron microscopy revealed that MYCi975 treatment induced alterations in the mitochondrial ultrastructure in MycCaP cells, characterized by electron-lucent matrices, severe cristae loss (cristolysis), and mitochondrial swelling (Fig. 3B), suggesting that the observed mitochondrial defects arise indirectly from MYCi975's broad transcriptional control of nuclear-encoded mitochondrial genes, rather than from a direct inhibition of complex I (*15*, *37*).

To assess the functional consequences of these morphological changes, we performed a real-time analysis of cellular bioenergetics using Seahorse XF technology in MycCaP cells. MYCi975 treatment caused a dose-dependent decrease in oxygen consumption rate (OCR), with maximal respiration reduced by over 75% at 8 μM (Fig. 3, C and E). This impairment in OXPHOS was accompanied by compromised glycolysis, as reflected by a substantial decrease in the extracellular acidification rate (ECAR) (Fig. 3, D, F, and G) and loss of glycolytic reserve (Fig. 3H). These data indicate that MYC inhibition simultaneously diminishes both major energy-producing pathways, leaving the cells unable to compensate energetically. Prompted by the synergy between Sod2 loss and MYC inhibition, we monitored ROS levels in MycCaP cells. As anticipated, MYCi975 elicited a dose-dependent increase in ROS levels (Fig. 3I), consistent with compromised mitochondrial function and reduced antioxidant capacity.

To better understand the transcriptional basis for these bioenergetic changes, we conducted RNA sequencing (RNA-seq) profiling in MycCaP cells treated with increasing MYCi975 concentrations and categorized the differentially expressed mitochondrial genes into distinct clusters (Fig. 3, J and K, and table S4). Cluster 4 genes, which include key components of mitochondrial translation, production, and maintenance, were repressed in a dose-dependent manner by MYCi975, aligning with MYC's known role in driving nuclear-encoded mitochondrial genes. Conversely, a subset of genes in cluster 3, enriched for complex I subunits and OXPHOS factors, was paradoxically up-regulated at 5 μM MYCi975 and inhibited at 10 μM, suggesting a possible compensatory attempt to maintain mitochondrial function. Notably, genes involved in mtDNA repair (cluster 2)

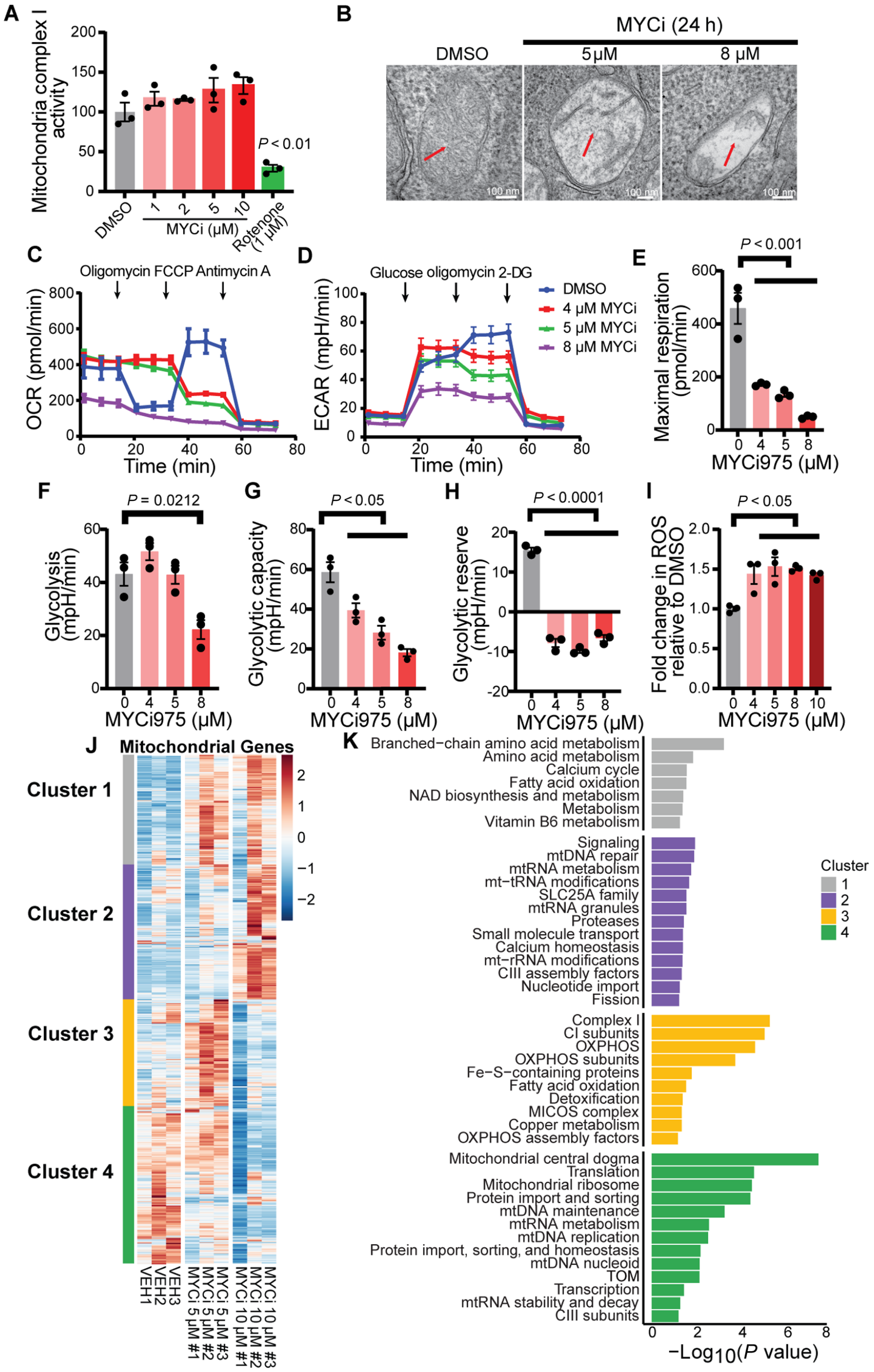

Fig. 3. MYC inhibition disrupts mitochondrial structure and cellular bioenergetics. (**A**) Complex I activity in isolated mitochondria exposed to MYCi975 (1 to 10 μM) or rotenone (1 μM); means ± SEM ($n = 3$). (**B**) Transmission electron microscopy of MycCaP cells treated with DMSO or MYCi975 (5 and 8 μM) for 24 hours. Red arrows mark dose-dependent cristae loss and matrix swelling. Scale bars, 100 nm. (**C**) Seahorse XF analysis of oxygen consumption rate (OCR) in MycCaP cells treated 24 hours with MYCi975, followed by sequential oligomycin (1 μM), carbonyl cyanide *p*-trifluoromethoxyphenylhydrazone (FCCP; 1 μM), and antimycin A (1 μM); means ± SEM ($n = 3$). (**D**) Extracellular acidification rate (ECAR) in MycCaP cells treated with indicated concentrations of MYCi975 for 24 hours. Sequential addition of glucose (10 mM), oligomycin (1 μM), and 2-DG (50 mM); means ± SEM ($n = 3$). (**E**) Maximal respiration quantified from OCR ($n = 3$). (**F** to **H**) Quantification of glycolysis (F), glycolytic capacity (G), and glycolytic reserve (H) from ECAR measurements ($n = 3$). (**I**) Relative ROS levels in MYCi975-treated MycCaP cells ($n = 3$). (**J**) Heatmap of mitochondrial gene expression after escalating MYCi975; four *z*-score clusters identified. (**K**) Gene-ontology enrichment of those clusters: cluster 4 (mitochondrial biogenesis/translation) is down-regulated; cluster 3 (complex I/OXPHOS) shows compensatory up-regulation at moderate inhibition; cluster 2 is enriched for mtDNA-repair genes. Bars indicate $-\log_{10}P$. Unless noted otherwise, significance was tested by one-way ANOVA with Tukey's post hoc comparison. NAD, nicotinamide adenine dinucleotide; mt-rRNA, mitochondrial ribosomal RNA; mtRNA, mitochondrial RNA; mtDNA, mitochondrial DNA; MICOS, mitochondrial contact site and cristae organizing system.

were also induced at higher levels of MYC inhibition, reflecting an attempt to preserve the mitochondrial genome integrity (Fig. 3, J and K).

Overall, these data show that partial MYC inhibition leads to both the down-regulation of core mitochondrial processes and up-regulation of compensatory ETC components, specifically complex I. When complex I is further compromised by genetic disruption or other means, this adaptive response fails, resulting in metabolic collapse and cell death. This mechanism could explain the synthetic lethal interaction between MYC inhibition and mitochondrial dysfunction, underscoring complex I as a critical vulnerability in MYC-driven cancers (*15*).

MYC inhibitors synergize with mitochondrial complex I inhibition in vitro and in vivo

Given our finding that complex I dysfunction is a critical vulnerability in MYC-inhibited cells, we wanted to test whether the Food and Drug Administration (FDA)–approved drug metformin, which inhibits complex I, enhances the efficacy of MYC-targeting agents. In MycCaP castration-resistant prostate cancer and LLC1 Lewis lung carcinoma cells, single-agent treatment with MYCi975 (4 μM in MycCaP and 3 μM in LLC1) or metformin (0.625 mM) produced minimal growth inhibition, whereas combinatorial treatment resulted in robust synergistic cytotoxicity (Fig. 4, A and B). This synergy was dependent on complex I function, as the reintroduction of yeast NDI1 reversed the dual-agent cytotoxic effects (Fig. 4C). This rescue experiment provides strong evidence mitochondrial complex I inhibition rather than the pleiotropic effects of metformin contributes to the synergy observed in these cells (*24*). Moreover, NDI1 also rescued the synergy between metformin and OmoMYC (Fig. 4D), indicating that complex I–based synergy extends beyond small-molecule MYC inhibitors and possibly involves a general mechanism of MYC inhibition. To further establish the extent of this phenomenon, we performed detailed Bliss synergy analyses in MycCaP, LLC1, and human prostate cancer cell lines (LNCaP, PC3, and 22Rv1) using a range of MYCi975 and metformin concentrations (fig. S3, A to E). Cytotoxicity synergy was observed across models, suggesting the broad applicability of this combination strategy.

Next, we investigated the mechanistic basis for this synergy by examining cellular energetics. Adenosine 5′-triphosphate (ATP) measurements revealed that, while MYCi975 and metformin each moderately reduced ATP levels when used alone, their combinatorial use caused a significant energetic collapse ($P < 0.0001$) (fig. S3F). NDI1 expression specifically rescued ATP levels in dual-treated cells ($P = 0.0041$), providing direct evidence that energetic collapse underlies the observed synthetic lethality between MYC and complex I inhibition (*38*).

We next examined the relationship between endogenous MYC expression and metformin sensitivity by analyzing the PRISM drug-repurposing dataset, focusing on cell lines at the extremes of MYC expression (top and bottom fifth percentiles, $n = 22$ to 24 cell lines total per drug) (*39*). This focused analysis of the expression extremes was chosen to maximize our ability to detect genuine biological relationships, while minimizing noise from intermediate expression levels. Both metformin and phenformin displayed significantly enhanced efficacy in MYC-low cells ($P = 0.0057$ and $P = 0.0019$, respectively) (fig. S4A) (*39*). Detailed examination of individual cell line responses across diverse cancer types revealed a notable pattern: cell lines with lower MYC expression consistently showed heightened sensitivity to complex I inhibition, regardless of cancer origin (fig. S4B and table S5). These findings provide independent validation that reduced MYC activity sensitizes cells to complex I inhibition, consistent with our findings that pharmacological MYC inhibition synergizes with metformin.

Last, we tested whether the synergy extended to in vivo tumor models. In MycCaP tumor-bearing mice, 14 days of single-agent MYCi975 [50 mg/kg, bis in die (BID)] or metformin (250 mg/kg, continuous administration) modestly inhibited tumor growth. In contrast, the combination treatment strongly suppressed tumor growth (Fig. 4E). A similar effect was observed in LLC1 tumor–bearing mice (fig. S4C). Collectively, these results highlight the translational potential of combining MYC inhibition with metformin and underscoring mitochondrial complex I as a promising target for MYC-directed therapies.

Metabolomic profiling reveals immunomodulatory effects of MYCi975 and metformin combination

To investigate how concurrent MYC and mitochondrial inhibition shapes tumor metabolism, we performed untargeted metabolomic profiling of MycCaP tumors harvested after 7 days of treatment with vehicle, MYCi975, metformin, or their combination. In line with our efficacy data (Fig. 4E and fig. S4C), the combination treatment markedly reduced tumor weight compared to the single agents (fig. S5A). Global metabolomic analysis revealed two major alterations: First, we detected a broad reduction in acylcarnitine species in combination-treated tumors (fig. S5B), which is often linked to mitochondrial dysfunction in cancer (*40*, *41*). This decrease reinforces the notion that MYCi975 and metformin act synergistically to impair mitochondrial metabolism (*40*, *41*). Notably, the individual treatments generated distinct signatures: MYCi975 modulated several metabolites such as gadobenic acid and nonadeca-10,13-dienoylcarnitine (fig. S5C), whereas metformin monotherapy was characterized by a marked enrichment of 1,1-dimethylbiguanide (metformin), confirming effective drug delivery into tumors (fig. S5D). Second, pathway-level analysis highlighted a notable increase in purine catabolism in the combination-treated tumors (Fig. 5, A and B, and table S6). Most prominently, uric acid, the terminal product of purine breakdown, was elevated (Fig. 5, A and B) (*42*). As purine metabolites have previously been implicated in regulating immune cell function (*43*, *44*), we next conducted an unbiased analysis focusing on seven metabolites present in both our untargeted metabolomics dataset and the Cancer Atlas of Metabolic Profiles (CAMP) database. Among these, uric acid showed the strongest correlation with immune populations in human prostate cancer (PRAD). High uric acid levels correlated positively with multiple antitumor immune subsets [$CD8^+$ T cells, natural killer (NK) cells, and macrophages] but inversely with immunosuppressive populations (regulatory T cells and eosinophils) (Fig. 5C and table S7, $n = 91$ human PRAD tumors). Similar trends were observed for the other malignancies in the CAMP database (Fig. 5D and table S7, $n = 550$ human tumors).

To validate these correlative findings in vivo, we performed flow cytometry on MycCaP tumors to assess immune infiltration. The combination treatment markedly enhanced the infiltration of multiple antitumor immune populations, specifically $CD8^+$ T cells, $CD45^+$ $F4/80^+$ macrophages, and $CD45^+$ $CD11c^+$ $CD11b^+$ dendritic cells, compared to the vehicle (Fig. 5E). Although other cell populations, including $CD45^+$ $CD11b^+$ $Gr1^+$ MDSCs, $CD3^+$ $CD49b^+$ $NK1.1^+$ APC^+ NK cells, and $CD4^+$ $CD25^+$ $Foxp3^+$ regulatory T cells, were also altered, these differences were not statistically significant (fig. S5E). In summary, these data suggest that convergent metabolic defects induced by dual MYC and complex I inhibition can amplify antitumor

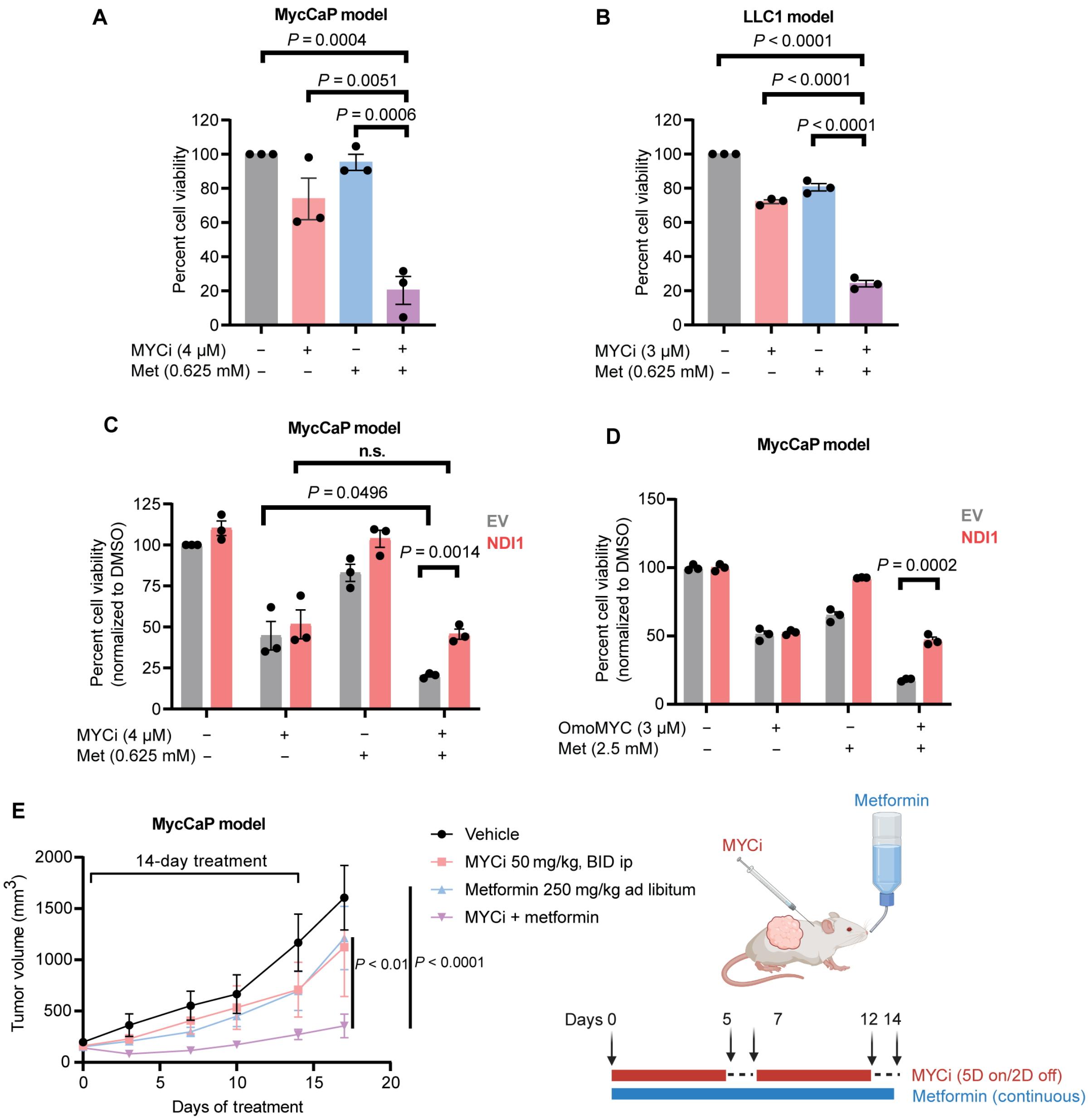

Fig. 4. MYC inhibitors synergize with metformin in preclinical cancer models. (**A** and **B**) Cell viability analysis showing synergistic effects of MYCi975 with metformin in MycCaP (A) and LLC1 (B) cells (data represent means ± SEM, $n = 3$; one-way ANOVA, Tukey's test). (**C** and **D**) NDI1 expression selectively rescues combination toxicity in MycCaP cells treated with MYCi975 (C) or OmoMYC (D) plus metformin, cell counting (data are means ± SEM, $n = 3$; unpaired Student's *t* test). Data in (A) to (D) are normalized to vehicle (DMSO). n.s., not significant. (**E**) Left: Schematic of the in vivo treatment regimen in MycCaP tumor-bearing mice, receiving MYCi975 by intraperitoneal injection (50 mg/kg, BID) on a 5-day on/2-day off (5D on/2D off) schedule and metformin by continuous oral administration (250 mg/kg per day). Right: Tumor volume curves over 14 days for vehicle ($n = 7$), MYCi975 ($n = 8$), metformin ($n = 10$), or combination ($n = 12$). Data are means ± SEM significance was assessed by unpaired two-tailed Student's *t* test. ip, intraperitoneally.

immunity, possibly by reshaping purine metabolism and elevating uric acid. This immunostimulatory facet may represent an additional therapeutic benefit of combining MYC inhibition with metformin.

DISCUSSION

Our study revealed a notable synthetic lethal interaction between MYC inhibition and mitochondrial metabolism, opening a promising therapeutic avenue for enhancing the effectiveness of emerging MYC inhibitors. Through an unbiased genome-wide CRISPR screen, we found that disruption of mitochondrial complex I creates a unique vulnerability in MYC-inhibited cells. This observation is especially pertinent given the recent progress in developing direct MYC inhibitors, which, despite overcoming a long-standing perception of MYC as an "undruggable" target, still lack established combination strategies to optimize their impact. By identifying mitochondrial metabolism as a key determinant of the MYC inhibitor response, our work addresses this critical knowledge gap (*26*).

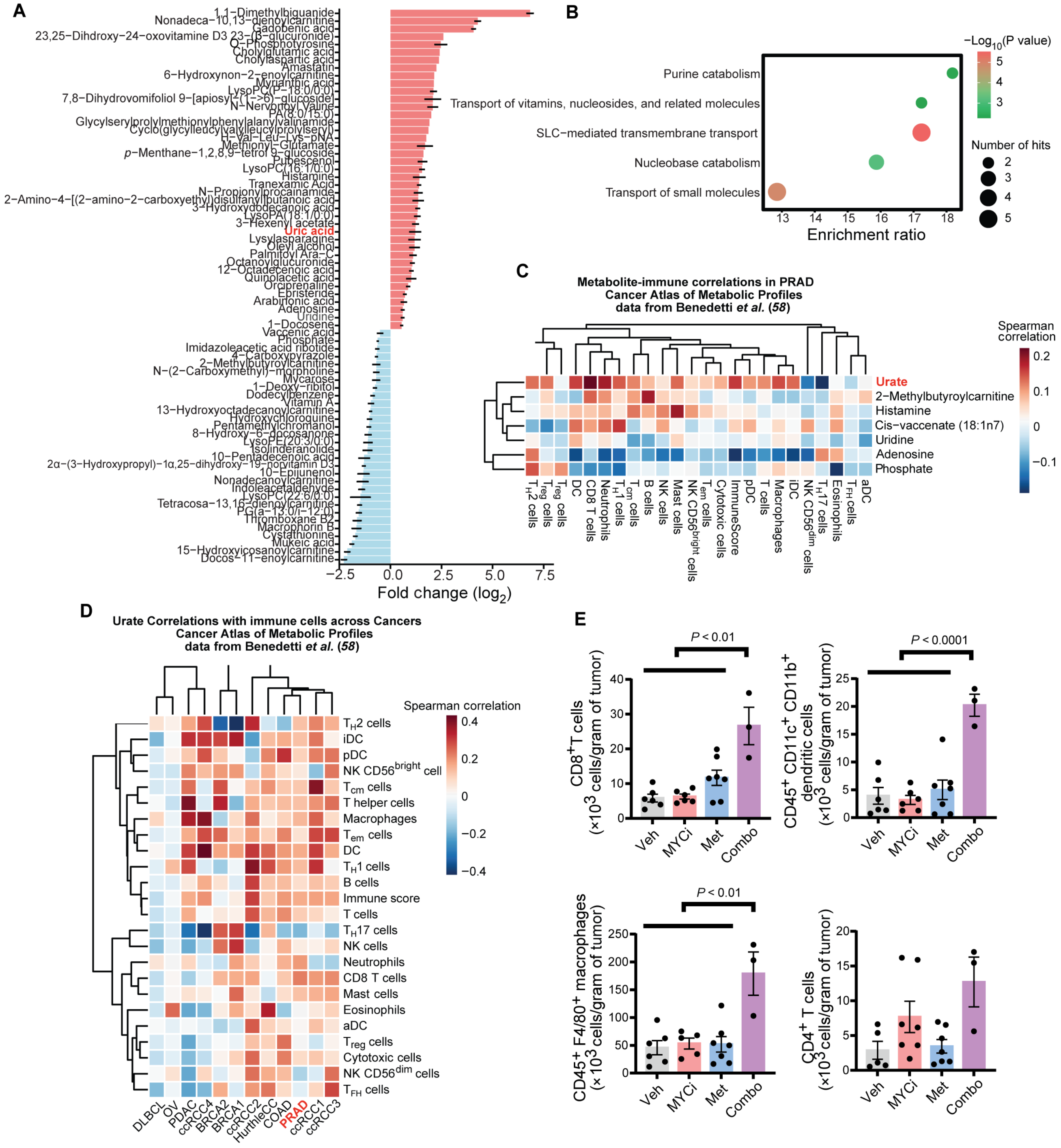

Fig. 5. Combined MYC and complex I inhibition reshapes tumor metabolism and immune landscape. (**A**) Differential metabolite abundance in MycCaP tumors treated with combination versus vehicle. Tumors were harvested after 7 days of treatment (vehicle, $n = 6$; MYCi, $n = 6$; metformin, $n = 5$; combination, $n = 5$; $\log_2$ fold change; data are means ± SEM). (**B**) Pathway enrichment analysis revealing significant up-regulation of purine catabolism and related transport pathways in combination-treated tumors. Circle size indicates number of metabolites; color intensity represents statistical significance [$-\log_{10}(P\text{ value})$]. SLC, solute carrier. (**C**) Correlation analysis between treatment-modulated metabolites and immune populations in human prostate cancer [prostate adenocarcinoma (PRAD)] from Cancer Atlas of Metabolic Profiles (CAMP) database ($n = 91$). Notably, uric acid shows strong positive correlation with antitumor immune subsets. Heatmap colors represent Spearman correlation coefficients. Data visualized by authors; metabolomic and immune-cell data from CAMP (*58*). (**D**) Pan-cancer analysis demonstrating consistent correlation between urate levels and immune cell populations across multiple cancer types, with particularly strong positive associations with $CD8^+$ T cells and macrophages. Heatmap colors represent Spearman correlation coefficients. Data visualized by authors; metabolomic and immune-cell data from CAMP (*58*). T_{FH} cells, T follicular helper cells; T_H1 cells, T helper 1 cells; T_H2 cells, T helper 2 cells; T_H17 cells, T helper 17 cells; T_{reg} cells, regulatory T cells. (**E**) Flow cytometry quantification of tumor-infiltrating immune cells following indicated treatments [$n = 3$ to 6 per group; data are means ± SEM; *P* values by one-way ANOVA (Tukey's)].

Mechanistically, this synthetic lethality is driven by the fact that MYC promotes both mitochondrial OXPHOS and glycolysis, thus providing cancer cells with high metabolic flexibility (*45*, *46*). When MYC is inhibited, particularly at moderate doses, glycolysis and overall mitochondrial function are both attenuated, but cells initially attempt to compensate by maintaining or even up-regulating some complex I subunits. This partial compensation keeps the cells alive but leaves them metabolically vulnerable. If complex I is further compromised (for example, by metformin), cells can no longer meet their energy demands through OXPHOS or fall back into sufficiently robust glycolysis. As a result, they undergo a profound "metabolic crisis," which leads to cell death. In this way, metformin's inhibition of complex I (*47*, *48*) synergizes with MYC inhibition to deplete the remaining respiratory capacity of MYC-inhibited cells, causing them to collapse under combined metabolic stress.

Furthermore, the identification of LDHA as a synthetic lethal hit complements our complex I findings, revealing how pyruvate metabolism influences MYC inhibitor sensitivity. When LDHA is inhibited, pyruvate is diverted toward mitochondria already compromised by MYC inhibition. This creates a metabolic bottleneck: MYC inhibition reduces both glycolysis and OXPHOS capacity, while LDHA inhibition forces increased mitochondrial pyruvate flux. This converging stress on mitochondrial metabolism explains the synthetic lethality observed with both LDHA and complex I targeting (*49*).

Another intriguing aspect of our findings is the connection between dual MYC/complex I disruption and tumor immune modulation. While our previous work established that MYC inhibition can trigger immunogenic cell death with release of damage-associated molecular patterns (*4*), our current data reveal a complementary metabolic mechanism. The combination treatment led to substantial alterations in purine metabolism, particularly an increase in uric acid levels and enhanced infiltration of immune cells. This observation suggests a potential dual mechanism: direct metabolic toxicity to cancer cells and facilitation of an immunostimulatory microenvironment (*50*, *51*). Rather than viewing these as competing explanations, we propose that they represent mutually reinforcing processes that collectively enhance antitumor immunity. Additional research is warranted to clarify how uric acid accumulation and related metabolic changes influence antitumor immune responses and whether these might be harnessed to extend the benefits of combined metabolic and immunotherapeutic strategies (*52*–*54*).

From a translational standpoint, our work demonstrated that metformin can synergize with MYC inhibitors to achieve significantly improved tumor control in vivo. Given its relatively safe profile, metformin has been tested in human cancers, albeit with limited success. Conversely, more potent complex I inhibitors suffer from unacceptable toxicity (*55*, *56*). Our results suggest that well-tolerated agents, such as metformin, could be combined with MYC inhibitors to achieve maximal clinical efficacy. In addition, our results suggest that baseline mitochondrial function or genetic signatures related to complex I expression may serve as predictive biomarkers to guide patient selection for MYC inhibitor therapy.

Although we focused specifically on metformin, it is possible that other FDA-approved or investigational metabolic modulators, such as phenformin or investigational complex I inhibitors, might also enhance the efficacy of MYC inhibitors. To translate these findings safely and effectively, future studies should carefully evaluate the potential toxicities associated with simultaneous MYC and mitochondrial disruption in normal tissues. Additionally, although our observations of immune modulation are encouraging, we have yet to fully elucidate the detailed immunological mechanisms underlying this synergy and whether other immunotherapies (e.g., checkpoint inhibitors) could augment it further. The tumor types examined here also do not encompass the full range of MYC-driven cancers, and it remains to be seen how broadly our findings may be applied to other malignancies. Last, although metformin is a widely used drug, optimal dosing schedules and the relative benefits of combining different MYC inhibitors with various metabolic agents require further preclinical and clinical studies.

In conclusion, we established that compromising mitochondrial metabolism, particularly complex I function, represents a key vulnerability factor in MYC-inhibited cancers. By demonstrating that approved metabolic agents, such as metformin, can exploit this vulnerability, our study provides a clear path toward clinically translatable regimens that bolster the efficacy of emerging MYC inhibitors. As direct MYC targeting continues to advance, leveraging metabolic dependencies may further expand therapeutic windows and offer fresh opportunities to integrate immunomodulatory strategies in the management of MYC-driven malignancies.

MATERIALS AND METHODS

Cell lines and reagents

MXycCaP (ATCC, no. CRL-3255, RRID:CVCL_J703) and LLC1 (ATCC, no. CRL-1642, RRID:CVCL_4358) cell lines were obtained from the American Type Culture Collection (ATCC) and maintained in Dulbecco's modified Eagle's medium (DMEM; Gibco, no. 11965118) supplemented with 10% fetal bovine serum (FBS; Thermo Fisher Scientific, no. A5256701), and 1% penicillin-streptomycin (Thermo Fisher Scientific, no. 15140122) at 37°C in a humidified atmosphere containing 5% CO_2. Human prostate cancer cell lines 22RV1 (ATCC, no. CRL-2505, RRID:CVCL_1045), LNCaP (ATCC, no. CRL-1740, RRID:CVCL_0395), and PC3 (ATCC, CRL-1435, RRID: CVCL_B0CB) were cultured in RPMI 1640 (Thermo Fisher Scientific, no. 118575119) supplemented with 10% FBS and 1% penicillin-streptomycin. Cell transfection was performed using Lipofectamine 2000 (Invitrogen, no. 11668-019) following the manufacturer's instructions, without additional antibiotics or supplements.

The small-molecule MYC inhibitor, MYCi975, was synthesized and characterized as previously described (*4*). For in vitro studies, MYCi975 was dissolved in dimethyl sulfoxide (DMSO) to prepare stock solutions. For in vivo administration, MYCi975 was formulated in 10% DMSO (MilliporeSigma, no. 472301), 20% Tween 80 (MP Biomedicals, no. 02194725.1), and 70% phosphate-buffered saline (PBS; Thermo Fisher Scientific no. 14-190-250). Metformin hydrochloride was obtained from Sigma-Aldrich (no. PHR1084) and was dissolved in sterile water.

For pyruvate metabolism studies, R-GNE-140 (LDHA inhibitor, MedChemExpress, no. HY-100742A), CPI-613 (PDH inhibitor, MedChemExpress, no. HY-15453), and PC-IN-1 (pyruvate carboxylase inhibitor, MedChemExpress, no. HY-142684) were dissolved in DMSO to prepare stock solutions. MitoTEMPO (MedChemExpress, no. HY-112879), a mitochondria-targeted antioxidant, was prepared as a 5 mM stock solution in sterile water.

The following antibodies were used for Western blotting and other analyses: anti-MYC (Abcam, no. ab32072, 1:1000, RRID:AB_731658), anti-NDUFA3 (OriGene Technologies, TA350213, 1:1000), anti-SOD2 (Cell Signaling Technology, no. 13141, 1:1000, RRID:AB_2636921),

and anti–β-actin (Cell Signaling Technology, no. 5125S, 1:5000, RRID:AB_1903890). The secondary antibodies included horseradish peroxidase (HRP)–conjugated anti-rabbit immunoglobulin G (IgG; Bio-Rad, no. 1706515, 1:2000, RRID:AB_11125142) and anti-mouse IgG (Bio-Rad no. 1706516, 1:2000, RRID:AB_2921252). All cell lines were authenticated using short tandem repeat profiling and routinely tested for mycoplasma contamination using MycoAlert (Lonza, no. LT07-418).

Whole genome CRISPR KO screening

A genome-wide CRISPR KO screen was conducted in MycCaP cells using the mTKO CRISPR library (Addgene, no. 159393, RRID:Addgene_159393), which targets 19,463 mouse genes with 94,528 sgRNAs (five sgRNAs per gene) (*27*, *57*). MycCaP cells were maintained in DMEM supplemented with 10% FBS and 1% penicillin-streptomycin at 37°C in 5% CO_2. Cells were transduced with the mTKO lentiviral library at a low multiplicity of infection (~0.3) and selected using puromycin (5 μg/ml) for 48 hours. The screen was performed in biological duplicates, with each replicate maintaining 400-fold coverage of the library (~3.8×10^7 cells per replicate). Three days after infection (t_0), cells were pooled and divided into treatment groups. Cells were treated with 3.5 μM MYCi975 (LD_{20}) or vehicle control for 9 days, equivalent to 10 population doublings, with cells replated every 3 days to maintain exponential growth.

Genomic DNA was extracted using the Wizard Genomic DNA Purification Kit (Promega, no. A1120), and sgRNA regions were amplified by two-step polymerase chain reaction (PCR) using the NEBNext Ultra II Q5 Master Mix (New England Biolabs, no. M0544L). First-round PCR was performed to enrich the guide RNA regions, followed by second-round PCR incorporating unique i5 and i7 multiplexing barcodes. The final gel-purified products were sequenced on an Illumina NextSeq 500 (RRID:SCR_014983) to determine sgRNA representation in each sample. The DrugZ algorithm was used to identify genes whose depletion led to enhanced sensitivity (synthetic lethal interactions) or resistance to MYCi975. For pathway enrichment analysis, synergistic hits were analyzed using PathfindR (RRID:SCR_025459), with GO annotations as the reference database. For each GO term, the fold enrichment scores and statistical significance were calculated. The top 15 enriched GO terms were visualized using ggplot2, with point colors representing $\log_{10}$-transformed *P* values and point sizes proportional to the number of genes mapped to each term (*29*).

RNA-seq analysis

MycCaP cells were treated with vehicle (DMSO), 5 μM MYCi975, or 10 μM MYCi975 for 18 hours in triplicate. Cells were harvested, and total RNA was extracted using the RNeasy Mini Kit (QIAGEN, no. 74134) according to the manufacturer's protocol. RNA quality and concentration were assessed using an Agilent 2100 Bioanalyzer, with all samples meeting the minimum RNA integrity number threshold of 9. RNA libraries were prepared, and library quality and size distribution were validated using Qubit fluorometric quantitation and an Agilent 2100 Bioanalyzer (RRID:SCR_018043). Sequencing was performed on an Illumina NextSeq500 platform using paired-end configuration.

Raw sequencing reads were aligned to the mouse genome (GRCm38/mm10) using STAR aligner (RRID:SCR_004463). Gene expression levels were quantified, and differential expression analysis was performed using DESeq2 (RRID:SCR_015687). For pathway analysis in MycCaP cells, mitochondrial genes were identified using the MitoCarta 3.0 database (RRID:SCR_018165) and clustered on the basis of their expression patterns. Mitochondrial pathway enrichment was determined using the MitoCarta-annotated pathways. For P493 and PC3 cells (GSE135800), pathway analysis was performed using GO terms with clusterProfiler (RRID:SCR_016884). Significantly enriched pathways were identified using an adjusted *P* value threshold of 0.05.

Mitochondrial complex gene analysis and donut plot

Mitochondrial respiratory chain complex components were identified using MitoCarta 3.0, with genes classified into their respective complexes (I to V). MYC-regulated genes were compiled from three sources: direct MYC targets from Morrish *et al.* (*14*) and differentially expressed genes from P493 and PC3 cells following MYC inhibition (RNA-seq data from this study). Synergistic genes were identified using genome-wide CRISPR screening. For cross-species analysis, mouse gene symbols were converted to human orthologs using the HGNC Comparison of Orthology Predictions database. The complete list of genes is provided in table S2. For visualization, a multilayer donut plot was constructed using the ggplot2 R package (RRID:SCR_014601).

siRNA-mediated knockdown

For MYC knockdown experiments, cells were seeded at 3×10^5 cells per well in six-well plates in antibiotic-free DMEM supplemented with 10% FBS. After 24 hours, MycCaP cells were transfected with ON-TARGETplus SMARTpool siRNA targeting MYC (Dharmacon, no. M-003282-07-0005) or a nontargeting control siRNA (Dharmacon, no. D-001210-01-05) using DharmaFECT 1 (Horizon Discovery, T-2001-02). For transfection, the siRNA was first diluted to working concentration of 5 μM in 1× siRNA buffer (Horizon Discovery, no. B-002000-UB-100). For each well, 10 μl of diluted siRNA was combined with 190 μl of serum-free medium (tube 1), and 10 μl of DharmaFECT 1 was diluted in 190 μl of serum-free medium (tube 2). After 5 min at room temperature, the contents of both tubes were combined and incubated for 20 min. The mixture was then supplemented with 1600 μl of antibiotic-free complete medium and added to the cells (final siRNA concentration, 25 nM; total volume, 2 ml per well). The culture medium was replaced with fresh complete medium 24 hours posttransfection. Cells were harvested 48 hours posttransfection for Western blot analysis to confirm the knockdown efficiency, followed by reseeding for subsequent experiments.

Complex I activity assay

Mitochondrial complex I activity was measured using a MitoCheck Complex I Activity Assay Kit (Cayman Chemical, no. 700930). Briefly, assay reagents were prepared in two tubes: tube A [910 μl of Complex I Activity Assay Buffer, 20 μl of KCN (1 mM), 50 μl of FF-Bovine Serum Albumin (FF-BSA) Assay Reagent, and 20 μl of Bovine Heart Mitochondria Assay Reagent] and tube B (625 μl of Complex I Activity Assay Buffer, 30 μl of NADH Assay Reagent, and 20 μl of Ubiquinone Assay Reagent). To measure the direct inhibition of complex I, isolated mitochondria were treated with increasing concentrations of MYCi975 (1 to 10 μM) or rotenone (1 μM) as a positive control. Assays were performed by adding 50 μl of tube A content, followed by 20 μl of test compound or vehicle, and initiated with 30 μl of tube B content. complex I activity was monitored by measuring NADH oxidation as a decrease in absorbance at 340 nm using a plate reader at 30-s intervals for 15 min at 25°C. Activity was

calculated relative to that of the vehicle control and expressed as a percentage of the control.

Electron microscopy

A total of 10,000 MycCaP cells grown on Thermanox plastic coverslips in 24-well plates were fixed in 0.1 M sodium cacodylate buffer (pH 7.35) containing 2% paraformaldehyde and 2.5% glutaraldehyde. The samples were postfixed with 2% osmium tetroxide in an unbuffered aqueous solution, rinsed with distilled water, and en bloc stained with 3% uranyl acetate. After rinsing with distilled water, the samples were dehydrated through ascending grades of ethanol, transitioned with a 1:1 mixture of ethanol and resin, and embedded in Embed 812 resin mixture. The samples were cured in a 60°C oven and sectioned on a Leica Ultracut UC6 ultramicrotome (RRID:SCR_020226). Thin sections (70 nm) were collected on 200 mesh copper grids and poststained with 3% uranyl acetate and Reynolds's lead citrate. Images were acquired using a FEI Tecnai Spirit G2 transmission electron microscope.

TCGA data analysis

For correlation analysis between MYC and complex I gene expression, we used The TCGA PRAD dataset with matched proteomics and RNA-seq data. MYC protein levels were correlated with expression of each complex I component using Spearman's rank correlation. For survival analysis, we stratified 493 patients with PRAD on the basis of complex I gene expression signatures. Patients were classified as "complex I–high" if any complex I gene exhibited expression z-score > 2, and "complex I–low" if all complex I genes had z-scores ≤ 2. Progression-free survival was analyzed using Kaplan-Meier curves, and statistical significance was assessed with log-rank tests.

Cell viability and proliferation assays

Cell viability was assessed by manual cell counting and luminescence-based assays. For manual cell counting experiments, cells were seeded in six-well plates (10,000 to 50,000 cells per well) and treated with the vehicle control (DMSO), MYCi975, metformin, or their combination at the indicated concentrations. At designated time points, the cells were trypsinized, stained with trypan blue, and counted using a Countess II automated cell counter (Thermo Fisher Scientific, RRID:SCR_025370). Cell numbers were normalized to the vehicle control.

For luminescence-based measurements, cells were seeded in 96-well plates (100 to 1000 cells per well, depending on the cell line) and treated with the indicated compounds 24 hours after plating. Cell viability was measured using the CellTiter-Glo Luminescent Cell Viability Assay kit (Promega, no. G7571) according to the manufacturer's instructions.

ATP measurement

Cellular ATP levels were measured using a bioluminescence-based ATP Determination Kit (Invitrogen, no. A22066). MycCaP cells were seeded in white-walled 96-well plates (5000 cells per well) and treated with MYCi975 (5 μM), metformin (1.25 mM), both agents, or vehicle control (DMSO) for 24 hours. For NDI1 rescue experiments, cells expressing empty vector or NDI1 were similarly treated. Following treatment, culture medium was removed, and cells were lysed with 50 μl of cell lysis buffer. Standard reaction solution (50 μl) containing D-luciferin, firefly luciferase, dithiothreitol, and reaction buffer was added to each well according to manufacturer's instructions. Luminescence was measured using a microplate reader. ATP concentrations were calculated using a standard curve generated with known ATP concentrations (1 nM to 1 μM).

DepMap AVANA database analysis

To investigate the relationship between Complex I expression and MYC dependency, we analyzed the DepMap AVANA-CRISPR dependency database (RRID:SCR_017655) (*35*). Gene dependency was measured using CERES scores, with scores less than −0.6 indicating strong dependency. For each cell line, the complex I expression score was calculated as follows

$$\text{Complex I score}_i = \frac{1}{n}\sum_{j=1}^{n} \text{gene}_j \tag{1}$$

where $n = 66$ complex I genes, including the respiratory chain components and assembly factors. The cell lines were classified into complex I–low (bottom 10th percentile, $n = 90$) and complex I–high (top 10th percentile, $n = 109$) groups.

For differential dependency analysis, delta dependency scores were calculated as

$$\text{Delta Score}_{\text{gene}} = \overline{\text{CERES}}_{\text{low}} - \overline{\text{CERES}}_{\text{high}} \tag{2}$$

where $\overline{\text{CERES}}_{\text{low}}$ and $\overline{\text{CERES}}_{\text{high}}$ represent the mean CERES scores in the complex I–low and complex I–high groups, respectively. Negative delta scores indicate stronger dependency in complex I–low cells. Statistical significance was assessed using a two-sided Wilcoxon rank sum test. Only genes with ≥3 valid measurements per group were included.

PRISM drug repurposing analysis

Drug sensitivity data and cell line expression profiles were obtained from the PRISM Repurposing dataset (2024 Q2 release) through the Broad Institute's DepMap portal (https://depmap.org/portal/data_page/?tab=allData&releasename=PRISM+Repurposing+Public+24Q2&filename=Repurposing_Public_24Q2_Extended_Primary_Data_Matrix.csv) (*39*). The expression data were log-transformed transcripts per million (TPM) values that were batch-corrected. Cell lines were stratified based on MYC expression levels, with the top fifth percentile classified as MYC-high ($n = 76$) and the bottom fifth percentile as MYC-low ($n = 76$). Drug sensitivity scores for metformin and phenformin were compared between the MYC-high and MYC-low groups where data are available. Statistical significance was assessed using a two-sided Student's t test, with outliers removed using an interquartile range threshold of 1.5. Cell lines without paired expression and sensitivity data were excluded from analysis. Individual cell line sensitivity scores were visualized using waterfall plots, with cell lines ordered according to increasing drug sensitivity. The results are provided in the tabular format in table S5.

CAMP data mining analysis

Metabolite-immune cell correlations were obtained from the CAMP database. Metabolite signatures were extracted from table S5 of the CAMP dataset (https://static-content.springer.com/esm/art%3A10.1038%2Fs42255-023-00817-8/MediaObjects/42255_2023_817_MOESM2_ESM.xlsx) (*58*), with a focus on seven metabolites of interest: 2-methylbutyroylcarnitine, adenosine, histamine, phosphate, uridine, cis-vaccenate (18:1n7), and urate. Correlations between

these metabolites and 24 immune cell populations were analyzed across multiple cancer types, with a focus on PRAD. For urate-specific analysis, correlations were examined across 13 tumor types. Hierarchical clustering was performed using Ward's method (ward. D2) with Manhattan distance. Pathway enrichment analysis was conducted on the differentially abundant metabolites using standard metabolic pathway databases, with enrichment ratios and *P* values calculated for each pathway. The results were visualized using heat maps generated using the pheatmap R package.

CRISPR-Cas9 KO

Individual gene KOs were generated using lentiviral delivery of sgRNAs in the plentiGuide vector targeting *Ndufa3* (sgNdufa3-1: 5′-GAGAAGGACACCACCAGCAC-3′; sgNdufa3-2: 5′-TTGGACCCAGAGGATCCTGA-3′) or *Sod2* (sgSod2-1: 5′-CTCGTGGTACTTCTCCTCGG-3′; sgSod2-2: 5′-TAGGCCTTATTCCGCTGCTG-3′). Lentiviral particles were produced in human embryonic kidney 293T cells by cotransfecting the sgRNA-expressing vector with the packaging plasmids psPAX2 and pMD2.G using calcium phosphate, as described previously (*57*). Viral supernatants were collected 48 hours posttransfection, filtered through 0.45-μm filters (VWR, no. 28145-481), and used to infect MycCaP-Cas9 cells in the presence of polybrene (8 μg/ml). Infected cells were selected with puromycin (5 μg/ml) for 5 days beginning 48 hours postinfection. KO efficiency was validated by immunoblotting using antibodies against NDUFA3 (OriGene Technologies, no. TA350213) and SOD2 (Cell Signaling Technology, no. 13141).

Western blotting

Protein lysates were prepared using radioimmunoprecipitation assay buffer supplemented with protease and phosphatase inhibitors. The protein concentration was determined using the BCA assay (Thermo Fisher Scientific, no. 23227). Lysates were loaded onto Bolt MOPs running buffer and transferred onto polyvinylidene difluoride membranes. Membranes were blocked with 5% milk in Tris-buffered saline with Tween 20 detergent (TBST) and incubated with primary antibodies overnight at 4°C. After washing, the membranes were incubated with HRP-conjugated secondary antibodies and visualized using the SuperSignal West Femto Maximum Sensitivity Substrate (Thermo Fisher Scientific, no. 34096).

Seahorse XF analysis

Cellular bioenergetics were analyzed using a Seahorse XF96 Extracellular Flux Analyzer (Agilent Technologies, RRID:SCR_019545) with both the XF Cell Mito Stress Test Kit (Agilent, no. 103015-100) and Glycolysis Stress Test Kit (Agilent, no. 103017-100). MycCaP and LLC1 cells were seeded at 10,000 cells per well in XF96 cell culture microplates (Agilent, no. 102416-100). After 24 hours, the cells were subjected to either mitochondrial or glycolytic analysis. For mitochondrial function, the growth medium was replaced with XF DMEM (pH 7.4) supplemented with 1 mM pyruvate, 2 mM glutamine, and 10 mM glucose. The cells were treated with MYCi975 (4 to 8 μM) for 24 hours before analysis. The OCR was measured under basal conditions, followed by sequential injection of oligomycin (1 μM), carbonyl cyanide *p*-trifluoromethoxyphenylhydrazone (1 μM), and a mixture of rotenone/antimycin A (1 μM each).

For glycolytic function, the medium was replaced with glucose-free XF-DMEM (pH 7.4) supplemented with 2 mM glutamine. The ECAR was measured at baseline, followed by sequential injections of glucose (10 mM), oligomycin (1 μM), and 2-deoxyglucose (50 mM). For both assays, the sensor cartridge was hydrated overnight in XF calibrant at 37°C in a non-CO_2 incubator. Data were acquired and analyzed using Wave software (Agilent, RRID:SCR_014526), with all measurements normalized to the cell number.

In vivo tumor models

All animal studies were conducted in accordance with protocols approved by the Institutional Animal Care and Use Committee of Northwestern University. Male C57BL/6 mice (6 to 8 weeks old) were subcutaneously injected with 1×10^6 MycCaP or LLC1 cells in a 1:1 mixture of PBS and Matrigel (Corning, no. 354234). Tumors were allowed to grow to ~200 mm^3 before randomization into the treatment groups.

For the MycCaP model, MYCi975 was administered intraperitoneally at 50 mg/kg twice daily (5 days on and 2 days off) for 14 days. Metformin was continuously administered in drinking water at 250 mg/kg per day for 14 days. In the LLC1 model, MYCi975 was administered for 10 days with a 2-day drug holiday, and metformin was administered continuously for 12 days. Tumors were measured biweekly using digital calipers and tumor growth inhibition was calculated. For immunophenotyping and metabolomics studies, mice were continuously treated with MYCi975 and metformin for 7 days, and tumors were harvested for analysis.

Flow cytometry analysis

Tumor samples were harvested after 7 days of treatment and processed immediately for flow cytometry analysis. Single-cell suspensions were prepared by mechanical dissociation of the tumor tissue using the Mouse Tumor Dissociation Kit (Miltenyi Biotec, no. 130-096-730, RRID:SCR_020285) according to the manufacturer's protocol. After dissociation, the samples were filtered through 70-μm cell strainers and treated with ACK lysis buffer (Lonza, no. 10-548E) to remove red blood cells. The cells were blocked with anti-mouse CD16/CD32 (BD Biosciences, no. 553142) to prevent nonspecific antibody binding.

For immunophenotyping, cells were stained with fluorochrome-conjugated antibodies against CD45 (BioLegend, no. 304038, RRID:AB_2562050), CD8 (eBioscience, no. 14-0808-82, RRID:AB_2572861), CD4 (BD Bioscience, no. 558107, RRID:AB_397030), CD11b (Novus Biologicals, no. NB110-89474SS, RRID:AB_1216360), CD11c (Thermo Fisher Scientific, no. 12-0114-82, RRID:AB_465552), F4/80 (BioLegend, no. 123124, RRID:AB_893475), Gr1 (BioLegend, no. 108401, RRID:AB_313366), CD49b (BioLegend, no. 108907, RRID:AB_313414), NK1.1 (BioLegend, no. 108709, RRID:AB_313396), and CD25 (Thermo Fisher Scientific, no. 25-0251-82, RRID:AB_469608). For intracellular Foxp3 staining, the cells were fixed and permeabilized using the Foxp3/Transcription Factor Staining Buffer Set (eBioscience, no. 88-8824-00). LIVE/DEAD Fixable Blue Dead Cell Stain (Thermo Fisher, no. L34962) was used to exclude dead cells. Samples were analyzed on a BD LSRFortessa flow cytometer, and data were processed using FlowJo software (v10.8.1). Cell numbers were normalized to the tumor weight and reported as cells per gram of tumor tissue.

Metabolomics analysis

Untargeted metabolomics was performed on snap-frozen tumor samples. Samples were prepared and analyzed using a Vanquish Flex Ultra High Performance Liquid Chromatography (UPLC) system coupled with a Q Exactive mass spectrometer (Thermo Fisher Scientific).

Metabolite extraction was performed by grinding tissue samples in 80% methanol, followed by sonication and centrifugation. The supernatants were analyzed by UPLC–tandem mass spectrometry. Metabolite data were processed using Compound Discoverer software, and multivariate statistical analysis was performed using SIMCA-P (version 14.1). Differential abundance analysis was performed between treatment groups (vehicle, MYCi975, metformin, and combination), with metabolites showing a fold change of >1.5 and $P < 0.05$, which was considered significant. For heatmap visualization, the metabolites were hierarchically clustered using Ward's method (ward.D2). Pathway analysis was performed using MetaboAnalyst 6.0 (RRID:SCR_015539), a web-based platform (www.metaboanalyst.ca).

Statistical analysis

Statistical analyses were performed using GraphPad Prism (version 9.0) (RRID:SCR_002798). For comparisons between two groups, unpaired two-tailed Student's t test was used. For multiple group comparisons, one-way analysis of variance (ANOVA) followed by Tukey's post hoc test was performed. For the drug combination studies, synergy was evaluated using the bliss independence model. For CAMP database analysis, correlations were evaluated using Spearman's correlation coefficients. Enrichment analysis P values were calculated using hypergeometric tests. Data are presented as means ± SEM unless otherwise indicated. Statistical significance is denoted as $*P < 0.05$, $**P < 0.01$, $***P < 0.001$, and $****P < 0.0001$.

Supplementary Materials

The PDF file includes:
Figs. S1 to S5
Legends for tables S1 to S7

Other Supplementary Material for this manuscript includes the following:
Tables S1 to S7

REFERENCES AND NOTES

1. M. Gabay, Y. Li, D. W. Felsher, MYC activation is a hallmark of cancer initiation and maintenance. *Cold Spring Harb. Perspect. Med.* **4**, a014241 (2014).
2. G. Donati, B. Amati, MYC and therapy resistance in cancer: Risks and opportunities. *Mol. Oncol.* **16**, 3828–3854 (2022).
3. V. Llombart, M. R. Mansour, Therapeutic targeting of "undruggable" MYC. *EBioMedicine* **75**, 103756 (2022).
4. H. Han, A. D. Jain, M. I. Truica, J. Izquierdo-Ferrer, J. F. Anker, B. Lysy, V. Sagar, Y. Luan, Z. R. Chalmers, K. Unno, H. Mok, R. Vatapalli, Y. A. Yoo, Y. Rodriguez, I. Kandela, J. B. Parker, D. Chakravarti, R. K. Mishra, G. E. Schiltz, S. A. Abdulkadir, Small-molecule MYC inhibitors suppress tumor growth and enhance immunotherapy. *Cancer Cell* **36**, 483–497.e15 (2019).
5. M. I. Truica, M. C. Burns, H. Han, S. A. Abdulkadir, Turning up the heat on MYC: Progress in small-molecule inhibitors. *Cancer Res.* **81**, 248–253 (2021).
6. H. Wang, J. Chauhan, A. Hu, K. Pendleton, J. L. Yap, P. E. Sabato, J. W. Jones, M. Perri, J. Yu, E. Cione, M. A. Kane, S. Fletcher, E. V. Prochownik, Disruption of Myc-Max heterodimerization with improved cell-penetrating analogs of the small molecule 10074-G5. *Oncotarget* **4**, 936–949 (2013).
7. M. Savino, D. Annibali, N. Carucci, E. Favuzzi, M. D. Cole, G. I. Evan, L. Soucek, S. Nasi, The action mechanism of the Myc inhibitor termed Omomyc may give clues on how to target Myc for cancer therapy. *PLOS ONE* **6**, e22284 (2011).
8. E. Garralda, M.-E. Beaulieu, V. Moreno, S. Casacuberta-Serra, S. Martínez-Martín, L. Foradada, G. Alonso, D. Massó-Vallés, S. López-Estévez, T. Jauset, E. C. de la Fuente, B. Doger, T. Hernández, R. Perez-Lopez, O. Arqués, V. C. Cano, J. Morales, J. R. Whitfield, M. Niewel, L. Soucek, E. Calvo, MYC targeting by OMO-103 in solid tumors: A phase 1 trial. *Nat. Med.* **30**, 762–771 (2024).
9. R. Dhanasekaran, A. Deutzmann, W. D. Mahauad-Fernandez, A. S. Hansen, A. M. Gouw, D. W. Felsher, The MYC oncogene - the grand orchestrator of cancer growth and immune evasion. *Nat. Rev. Clin. Oncol.* **19**, 23–36 (2022).
10. D. M. Miller, S. D. Thomas, A. Islam, D. Muench, K. Sedoris, c-Myc and cancer metabolism. *Clin. Cancer Res.* **18**, 5546–5553 (2012).
11. G. Bretones, M. D. Delgado, J. León, Myc and cell cycle control. *Biochim. Biophys. Acta* **1849**, 506–516 (2015).
12. E. S. Goetzman, E. V. Prochownik, The role for Myc in coordinating glycolysis, oxidative phosphorylation, glutaminolysis, and fatty acid metabolism in normal and neoplastic tissues. *Front. Endocrinol. (Lausanne)* **9**, 129 (2018).
13. C. V. Dang, MYC on the path to cancer. *Cell* **149**, 22–35 (2012).
14. F. Morrish, D. Hockenbery, MYC and mitochondrial biogenesis. *Cold Spring Harb. Perspect. Med.* **4**, a014225 (2014).
15. F. Li, Y. Wang, K. I. Zeller, J. J. Potter, D. R. Wonsey, K. A. O'Donnell, J.-W. Kim, J. T. Yustein, L. A. Lee, C. V. Dang, Myc stimulates nuclearly encoded mitochondrial genes and mitochondrial biogenesis. *Mol. Cell. Biol.* **25**, 6225–6234 (2005).
16. J. A. Graves, Y. Wang, S. Sims-Lucas, E. Cherok, K. Rothermund, M. F. Branca, J. Elster, D. Beer-Stolz, B. Van Houten, J. Vockley, E. V. Prochownik, Mitochondrial structure, function and dynamics are temporally controlled by c-Myc. *PLOS ONE* **7**, e37699 (2012).
17. S. Cermelli, I. S. Jang, B. Bernard, C. Grandori, Synthetic lethal screens as a means to understand and treat MYC-driven cancers. *Cold Spring Harb. Perspect. Med.* **4**, a014209 (2014).
18. R. M. Chabanon, S. Postel-Vinay, A novel synthetic lethal approach to target MYC-driven cancers. *Cancer Res.* **82**, 969–971 (2022).
19. X. G. Zhu, A. Chudnovskiy, L. Baudrier, B. Prizer, Y. Liu, B. N. Ostendorf, N. Yamaguchi, A. Arab, B. Tavora, R. Timson, S. Heissel, E. de Stanchina, H. Molina, G. D. Victora, H. Goodarzi, K. Birsoy, Functional genomics in vivo reveal metabolic dependencies of pancreatic cancer cells. *Cell Metab.* **33**, 211–221.e6 (2021).
20. M. Tang, S. O'Grady, J. Crown, M. J. Duffy, MYC as a therapeutic target for the treatment of triple-negative breast cancer: Preclinical investigations with the novel MYC inhibitor, MYCi975. *Breast Cancer Res. Treat.* **195**, 105–115 (2022).
21. A. G. Holmes, J. B. Parker, V. Sagar, M. I. Truica, P. N. Soni, H. Han, G. E. Schiltz, S. A. Abdulkadir, D. Chakravarti, A MYC inhibitor selectively alters the MYC and MAX cistromes and modulates the epigenomic landscape to regulate target gene expression. *Sci. Adv.* **8**, eabh3635 (2022).
22. W. W. Wheaton, S. E. Weinberg, R. B. Hamanaka, S. Soberanes, L. B. Sullivan, E. Anso, A. Glasauer, E. Dufour, G. M. Mutlu, G. S. Budigner, N. S. Chandel, Metformin inhibits mitochondrial complex I of cancer cells to reduce tumorigenesis. *eLife* **3**, e02242 (2014).
23. E. Fontaine, Metformin-induced mitochondrial complex I inhibition: Facts, uncertainties, and consequences. *Front. Endocrinol. (Lausanne)* **9**, 753 (2018).
24. A. S. Krall, P. J. Mullen, F. Surjono, M. Momcilovic, E. W. Schmid, C. J. Halbrook, A. Thambundit, S. D. Mittelman, C. A. Lyssiotis, D. B. Shackelford, S. R. V. Knott, H. R. Christofk, Asparagine couples mitochondrial respiration to ATF4 activity and tumor growth. *Cell Metab.* **33**, 1013–1026.e6 (2021).
25. S. K. Madden, A. D. de Araujo, M. Gerhardt, D. P. Fairlie, J. M. Mason, Taking the Myc out of cancer: Toward therapeutic strategies to directly inhibit c-Myc. *Mol. Cancer* **20**, 3 (2021).
26. J. R. Whitfield, L. Soucek, The long journey to bring a Myc inhibitor to the clinic. *J. Cell Biol.* **220**, e202103090 (2021).
27. K. A. Lawson, C. M. Sousa, X. Zhang, E. Kim, R. Akthar, J. J. Caumanns, Y. Yao, N. Mikolajewicz, C. Ross, K. R. Brown, A. A. Zid, Z. P. Fan, S. Hui, J. A. Krall, D. M. Simons, C. J. Slater, V. De Jesus, L. Tang, R. Singh, J. E. Goldford, S. Martin, Q. Huang, E. A. Francis, A. Habsid, R. Climie, D. Tieu, J. Wei, R. Li, A. H. Y. Tong, M. Aregger, K. S. Chan, H. Han, X. Wang, P. Mero, J. H. Brumell, A. Finelli, L. Ailles, G. Bader, G. A. Smolen, G. A. Kingsbury, T. Hart, C. Kung, J. Moffat, Functional genomic landscape of cancer-intrinsic evasion of killing by T cells. *Nature* **586**, 120–126 (2020).
28. T. Wang, K. Birsoy, N. W. Hughes, K. M. Krupczak, Y. Post, J. J. Wei, E. S. Lander, D. M. Sabatini, Identification and characterization of essential genes in the human genome. *Science* **350**, 1096–1101 (2015).
29. M. Colic, G. Wang, M. Zimmermann, K. Mascall, M. McLaughlin, L. Bertolet, W. F. Lenoir, J. Moffat, S. Angers, D. Durocher, T. Hart, Identifying chemogenetic interactions from CRISPR screens with drugZ. *Genome Med.* **11**, 52 (2019).
30. M. Rak, P. Rustin, Supernumerary subunits NDUFA3, NDUFA5 and NDUFA12 are required for the formation of the extramembrane arm of human mitochondrial complex I. *FEBS Lett.* **588**, 1832–1838 (2014).
31. F. R. Palma, C. He, J. M. Danes, V. Paviani, D. R. Coelho, B. N. Gantner, M. G. Bonini, Mitochondrial superoxide dismutase: What the established, the intriguing, and the novel reveal about a key cellular redox switch. *Antioxid. Redox Signal.* **32**, 701–714 (2020).
32. G. S. McElroy, C. R. Reczek, P. A. Reyfman, D. S. Mithal, C. M. Horbinski, N. S. Chandel, NAD+ regeneration rescues lifespan, but not ataxia, in a mouse model of brain mitochondrial Complex I dysfunction. *Cell Metab.* **32**, 301–308.e6 (2020).
33. L. Granat, D. Y. Knorr, D. C. Ranson, E. L. Hamer, R. P. Chakrabarty, F. Mattedi, L. Fort-Aznar, F. Hirth, S. T. Sweeney, A. Vagnoni, N. S. Chandel, J. M. Bateman, Yeast NDI1 reconfigures neuronal metabolism and prevents the unfolded protein response in mitochondrial complex I deficiency. *PLOS Genet.* **19**, e1010793 (2023).
34. S.-G. Yang, H.-J. Park, J.-W. Kim, J.-M. Jung, M.-J. Kim, H.-G. Jegal, I.-S. Kim, M.-J. Kang, G. Wee, H.-Y. Yang, Y.-H. Lee, J.-H. Seo, S.-U. Kim, D.-B. Koo, Mito-TEMPO improves

development competence by reducing superoxide in preimplantation porcine embryos. *Sci. Rep.* **8**, 10130 (2018).
35. A. Tsherniak, F. Vazquez, P. G. Montgomery, B. A. Weir, G. Kryukov, G. S. Cowley, S. Gill, W. F. Harrington, S. Pantel, J. M. Krill-Burger, R. M. Meyers, L. Ali, A. Goodale, Y. Lee, G. Jiang, J. Hsiao, W. F. J. Gerath, S. Howell, E. Merkel, M. Ghandi, L. A. Garraway, D. E. Root, T. R. Golub, J. S. Boehm, W. C. Hahn, Defining a cancer dependency map. *Cell* **170**, 564–576.e16 (2017).
36. S. Heinz, A. Freyberger, B. Lawrenz, L. Schladt, G. Schmuck, H. Ellinger-Ziegelbauer, Mechanistic investigations of the mitochondrial complex I inhibitor rotenone in the context of pharmacological and safety evaluation. *Sci. Rep.* **7**, 45465 (2017).
37. E. Agarwal, B. J. Altman, J. H. Seo, J. C. Ghosh, A. V. Kossenkov, H.-Y. Tang, S. R. Krishn, L. R. Languino, D. I. Gabrilovich, D. W. Speicher, C. V. Dang, D. C. Altieri, Myc-mediated transcriptional regulation of the mitochondrial chaperone TRAP1 controls primary and metastatic tumor growth. *J. Biol. Chem.* **294**, 10407–10414 (2019).
38. H. Wang, L. Sharma, J. Lu, P. Finch, S. Fletcher, E. V. Prochownik, Structurally diverse c-Myc inhibitors share a common mechanism of action involving ATP depletion. *Oncotarget* **6**, 15857–15870 (2015).
39. S. M. Corsello, J. A. Bittker, Z. Liu, J. Gould, P. McCarren, J. E. Hirschman, S. E. Johnston, A. Vrcic, B. Wong, M. Khan, J. Asiedu, R. Narayan, C. C. Mader, A. Subramanian, T. R. Golub, The drug repurposing hub: A next-generation drug library and information resource. *Nat. Med.* **23**, 405–408 (2017).
40. M. R. McCann, M. V. George De la Rosa, G. R. Rosania, K. A. Stringer, L-carnitine and acylcarnitines: Mitochondrial biomarkers for precision medicine. *Metabolites* **11**, 51 (2021).
41. E. Kankuri, P. Finckenberg, J. Leinonen, M. Tarkia, S. Björk, J. Purhonen, J. Kallijärvi, M. Kankainen, R. Soliymani, M. Lalowski, E. Mervaala, Altered acylcarnitine metabolism and inflexible mitochondrial fuel utilization characterize the loss of neonatal myocardial regeneration capacity. *Exp. Mol. Med.* **55**, 806–817 (2023).
42. A. Kushiyama, Y. Nakatsu, Y. Matsunaga, T. Yamamotoya, K. Mori, K. Ueda, Y. Inoue, H. Sakoda, M. Fujishiro, H. Ono, T. Asano, Role of uric acid metabolism-related inflammation in the pathogenesis of metabolic syndrome components such as atherosclerosis and nonalcoholic steatohepatitis. *Mediators Inflamm.* **2016**, 8603164 (2016).
43. J. E. Seegmiller, T. Watanabe, M. H. Shreier, T. A. Waldmann, Immunological aspects of purine metabolism. *Adv. Exp. Med. Biol.* **76A**, 412–433 (1977).
44. Z. Wu, Y. Gao, L. Cao, Q. Peng, X. Yao, Purine metabolism-related genes and immunization in thyroid eye disease were validated using bioinformatics and machine learning. *Sci. Rep.* **13**, 18391 (2023).
45. R. Shiratori, K. Furuichi, M. Yamaguchi, N. Miyazaki, H. Aoki, H. Chibana, K. Ito, S. Aoki, Glycolytic suppression dramatically changes the intracellular metabolic profile of multiple cancer cell lines in a mitochondrial metabolism-dependent manner. *Sci. Rep.* **9**, 18699 (2019).
46. R. J. DeBerardinis, N. S. Chandel, Fundamentals of cancer metabolism. *Sci. Adv.* **2**, e1600200 (2016).
47. S. R. Lord, A. L. Harris, Is it still worth pursuing the repurposing of metformin as a cancer therapeutic? *Br. J. Cancer* **128**, 958–966 (2023).
48. D. Bezwada, L. Perelli, N. P. Lesner, L. Cai, B. Brooks, Z. Wu, H. S. Vu, V. Sondhi, D. L. Cassidy, S. Kasitinon, S. Kelekar, F. Cai, A. B. Aurora, M. Patrick, A. Leach, R. Ghandour, Y. Zhang, D. Do, P. McDaniel, J. Sudderth, D. Dumesnil, S. House, T. Rosales, A. M. Poole, Y. Lotan, S. Woldu, A. Bagrodia, X. Meng, J. A. Cadeddu, P. Mishra, J. Garcia-Bermudez, I. Pedrosa, P. Kapur, K. D. Courtney, C. R. Malloy, G. Genovese, V. Margulis, R. J. DeBerardinis, Mitochondrial complex I promotes kidney cancer metastasis. *Nature* **633**, 923–931 (2024).
49. H. Shim, C. Dolde, B. C. Lewis, C. S. Wu, G. Dang, R. A. Jungmann, R. Dalla-Favera, C. V. Dang, c-Myc transactivation of LDH-A: Implications for tumor metabolism and growth. *Proc. Natl. Acad. Sci. U.S.A.* **94**, 6658–6663 (1997).
50. J. Linden, F. Koch-Nolte, G. Dahl, Purine release, metabolism, and signaling in the inflammatory response. *Annu. Rev. Immunol.* **37**, 325–347 (2019).
51. Z. Wu, D. Bezwada, F. Cai, R. C. Harris, B. Ko, V. Sondhi, C. Pan, H. S. Vu, P. T. Nguyen, B. Faubert, L. Cai, H. Chen, M. Martin-Sandoval, D. Do, W. Gu, Y. Zhang, Y. Zhang, B. Brooks, S. Kelekar, L. G. Zacharias, K. C. Oaxaca, J. S. Patricio, T. P. Mathews, J. Garcia-Bermudez, M. Ni, R. J. DeBerardinis, Electron transport chain inhibition increases cellular dependence on purine transport and salvage. *Cell Metab.* **36**, 1504–1520.e9 (2024).
52. S. Mi, L. Gong, Z. Sui, Friend or foe? An unrecognized role of uric acid in cancer development and the potential anticancer effects of uric acid-lowering drugs. *J. Cancer* **11**, 5236–5244 (2020).
53. C. M. Shachaf, A. M. Kopelman, C. Arvanitis, A. Karlsson, S. Beer, S. Mandl, M. H. Bachmann, A. D. Borowsky, B. Ruebner, R. D. Cardiff, Q. Yang, J. M. Bishop, C. H. Contag, D. W. Felsher, MYC inactivation uncovers pluripotent differentiation and tumour dormancy in hepatocellular cancer. *Nature* **431**, 1112–1117 (2004).
54. C. M. Shachaf, D. W. Felsher, Tumor dormancy and MYC inactivation: Pushing cancer to the brink of normalcy. *Cancer Res.* **65**, 4471–4474 (2005).
55. T. A. Yap, N. Daver, M. Mahendra, J. Zhang, C. Kamiya-Matsuoka, F. Meric-Bernstam, H. M. Kantarjian, F. Ravandi, M. E. Collins, M. E. D. Francesco, E. E. Dumbrava, S. Fu, S. Gao, J. P. Gay, S. Gera, J. Han, D. S. Hong, E. J. Jabbour, Z. Ju, D. D. Karp, A. Lodi, J. R. Molina, N. Baran, A. Naing, M. Ohanian, S. Pant, N. Pemmaraju, P. Bose, S. A. Piha-Paul, J. Rodon, C. Salguero, K. Sasaki, A. K. Singh, V. Subbiah, A. M. Tsimberidou, Q. A. Xu, M. Yilmaz, Q. Zhang, Y. Li, C. A. Bristow, M. B. Bhattacharjee, S. Tiziani, T. P. Heffernan, C. P. Vellano, P. Jones, C. J. Heijnen, A. Kavelaars, J. R. Marszalek, M. Konopleva, Complex I inhibitor of oxidative phosphorylation in advanced solid tumors and acute myeloid leukemia: Phase I trials. *Nat. Med.* **29**, 115–126 (2023).
56. X. Zhang, C. V. Dang, Time to hit pause on mitochondria-targeting cancer therapies. *Nat. Med.* **29**, 29–30 (2023).
57. D. Gallo, J. T. F. Young, J. Fourtounis, G. Martino, A. Álvarez-Quilón, C. Bernier, N. M. Duffy, R. Papp, A. Roulston, R. Stocco, J. Szychowski, A. Veloso, H. Alam, P. S. Baruah, A. B. Fortin, J. Bowlan, N. Chaudhary, J. Desjardins, E. Dietrich, S. Fournier, C. Fugère-Desjardins, T. G. de Rugy, M.-E. Leclaire, B. Liu, V. Bhaskaran, Y. Mamane, H. Melo, O. Nicolas, A. Singhania, R. K. Szilard, J. Tkáč, S. Y. Yin, S. J. Morris, M. Zinda, C. G. Marshall, D. Durocher, CCNE1 amplification is synthetic lethal with PKMYT1 kinase inhibition. *Nature* **604**, 749–756 (2022).
58. E. Benedetti, E. M. Liu, C. Tang, F. Kuo, M. Buyukozkan, T. Park, J. Park, F. Correa, A. A. Hakimi, A. M. Intlekofer, J. Krumsiek, E. Reznik, A multimodal atlas of tumour metabolism reveals the architecture of gene-metabolite covariation. *Nat. Metab.* **5**, 1029–1044 (2023).

Acknowledgments: We thank L. Reynolds Jr. from the Northwestern Center for Advanced Microscopy for helping with electron microscopy design, execution, and analysis. We thank C. V. Dang for providing the P493-6 B cells. We thank NUseq and Robert H. Lurie Comprehensive Cancer Center Flow Cytometry cores of Northwestern University. We thank all Abdulkadir lab members for valuable discussions. Figure 1A was created with BioRender.com. **Funding:** This work was supported by NIH, grants P50 CA180995 (S.A.A.) and R01 CA257258 (S.A.A. and D.C.); Prostate Cancer Foundation (PCF) TACTICAL Award (S.A.A.); and Polsky Urologic Cancer Institute (S.A.A.). **Author contributions:** Conceptualization: W.Y., H.T.P., D.C., and S.A.A. Methodology: W.Y., Q.G., S.Q., M.F.D., Z.R.C., J.B.P., K.V., H.T.P., D.C., and D.G.G. Investigation: W.Y., Q.G., S.Q., M.F.D., Z.R.C., J.B.P., K.V., M.T., M.M.K., D.G.G., A.W.T.S., H.P., M.S., H.T.P., G.E.S., D.C., and N.S.C. Formal analysis: W.Y., Z.R.C., J.B.P., and H.T.P. Validation: M.T., H.T.P., W.Y., D.G.G., and D.C. Resources: H.T.P., D.C., M.M.K., and D.G.G. Data curation: H.T.P., W.Y., and D.G.G. Software: H.T.P., W.Y., and D.G.G. Visualization: H.T.P., W.Y., and D.C. Project administration: W.Y. and M.M.K. Supervision: D.C. and S.A.A. Funding acquisition: D.C. and S.A.A. Writing—original draft: W.Y., H.T.P., D.C., and S.A.A. Writing—review and editing: all authors. **Competing interests:** S.A.A. and G.E.S. are coinventors on patents covering the methods and assays to identify and characterize MYC inhibitors and derivatives (US11420957B2, "Substituted heterocycles as c-MYC targeting agents," filed by Northwestern University, filed 17 June 2020 and published 23 August 2022). All other authors declare that they have no competing interests. **Data and materials availability:** The RNA-seq data from P493 and PC3 cells used in this study were obtained from the Gene Expression Omnibus (GEO) under accession number GSE135800. The PRISM Repurposing dataset (2024 Q2 release) was obtained from the DepMap portal of the Broad Institute (https://depmap.org/portal/). Metabolite-immune cell correlation data were obtained from the CAMP database (https://static-content.springer.com/esm/art%3A10.1038%2Fs42255-023-00817-8/MediaObjects/42255_2023_817_MOESM2_ESM.xlsx). Mitochondrial complex gene annotations were obtained using MitoCarta 3.0. All other data needed to evaluate the conclusions in the paper are present in the paper and/or the Supplementary Materials.

Submitted 6 February 2025
Accepted 11 June 2025
Published 16 July 2025
10.1126/sciadv.adw5228
