# Supplementary material for: Impaired mitochondrial metabolism is a critical cancer vulnerability for MYC inhibitors
Source: Sci Adv. 2025 Jul 16;11(29):eadw5228. doi: 10.1126/sciadv.adw5228 (PMC12266110; doi:10.1126/sciadv.adw5228)
Supplement: Supplementary file 2 — Figs. S1 to S5 Legends for tables S1 to S7 [file sciadv.adw5228_sm.pdf]

Science Advances
AAAS

Supplementary Materials for

Impaired mitochondrial metabolism is a critical cancer vulnerability for MYC inhibitors

William Yang *et al.*

Corresponding author: Sarki A. Abdulkadir, sarki.abdulkadir@northwestern.edu

Sci. Adv. **11**, eadw5228 (2025)
DOI: 10.1126/sciadv.adw5228

The PDF file includes:

Figs. S1 to S5
Legends for tables S1 to S7

Other Supplementary Material for this manuscript includes the following:

Tables S1 to S7

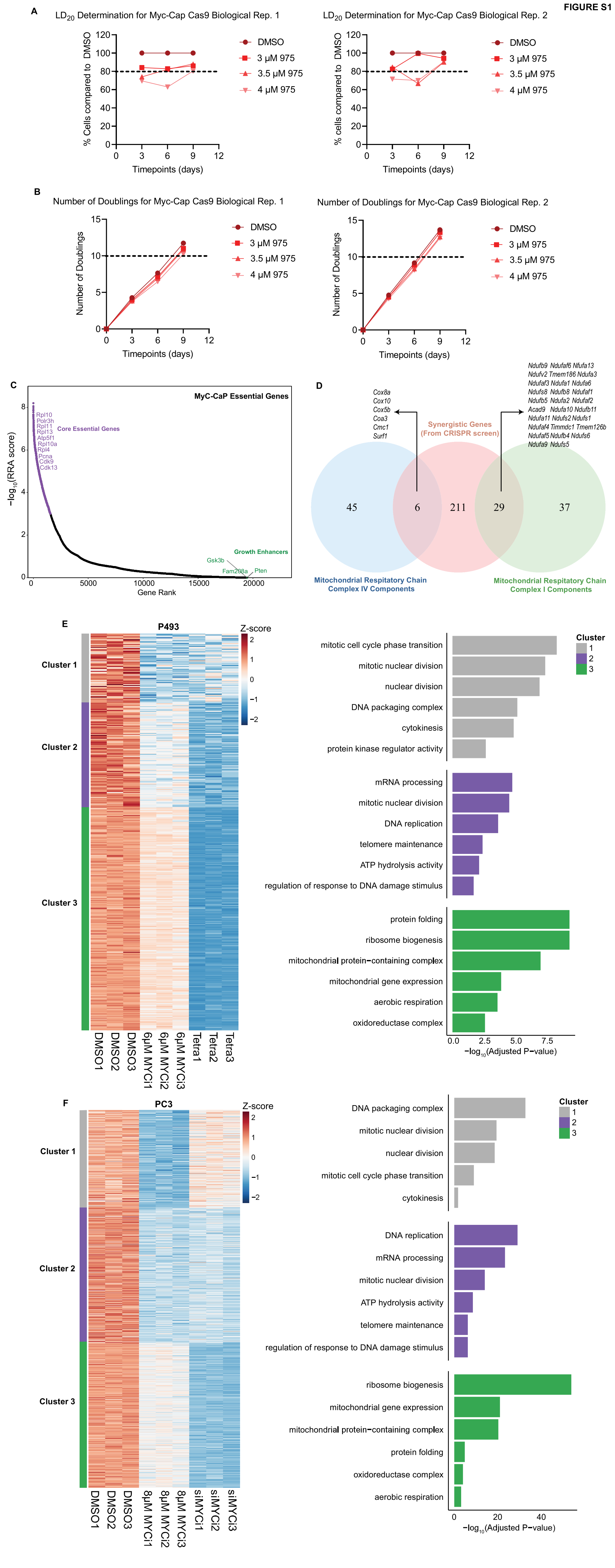

Figure S1. Validation of CRISPR-screen conditions and transcriptional profiling of MYC-regulated pathways.
(A) LD_{20} determination for MYCi975 in MycCaP Cas9 cells across two biological replicates. Cell viability was monitored over 12 days at indicated drug concentrations. Dashed line indicates 80% viability threshold. (**B)** Population doubling analysis confirming consistent achievement of 10 doublings within the 9-day treatment period across replicates and conditions. (**C)** Distribution of essential genes identified in MycCaP cells showing expected core fitness genes and growth enhancers. $-\log_{10}$(RRA score) plotted against gene rank. (**D)** Venn diagram overlapping the CRISPR screen hits with mitochondrial respiratory chain Complex I and IV components, showing significant enrichment for Complex I (29/66 genes). (**E and F)** RNA-seq analysis of P493 B-cell lymphoma (E) and PC3 prostate cancer (F) cells showing clustering of differentially expressed genes after MYC inhibition by MYCi975 for 24 hr, tetracycline-induced MYC repression (Tetra), or siRNA-mediated MYC knockdown (siMYC). Three distinct clusters emerge: clusters 1/2 show notable repression of cell cycle and DNA replication genes, while cluster 3 genes (ribosome biogenesis and mitochondrial metabolism) are partially preserved. This pattern is consistent across both cell contexts. Gene Ontology analysis shows pathway enrich-ment for each cluster (right panels).

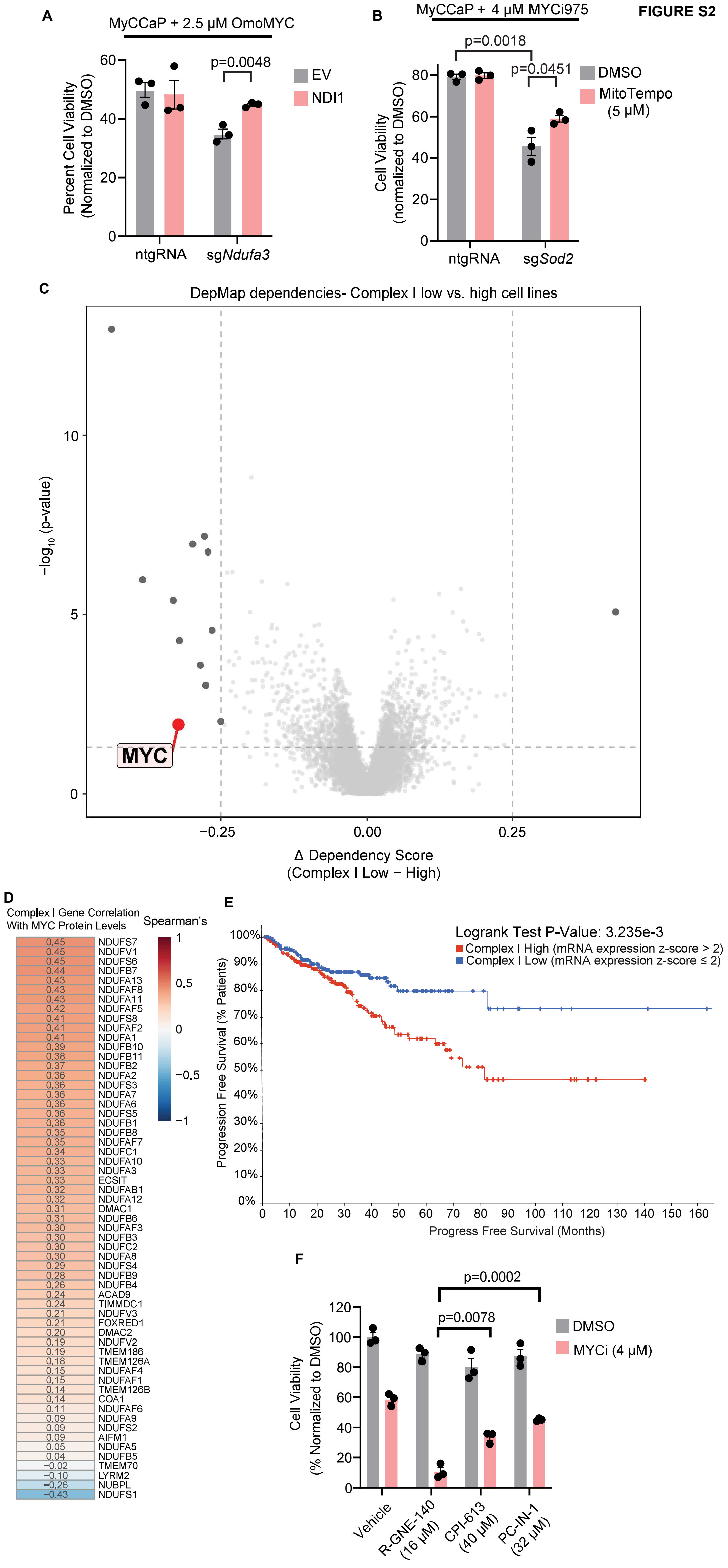

Figure S2. Validation of Complex I-dependent synthetic lethality.
(A) Cell viability of MycCaP cells expressing empty vector (EV) or NDI1 treated with 2.5 µM OmoMYC for 3 days. NDI1 expression rescues synthetic lethality specifically in Ndufa3-depleted cells. Data are mean ± SEM (n=3); unpaired two-tailed Student's t-test. **(B)** MitoTEMPO (5 µM) partially rescues synthetic lethality between SOD2 loss and MYC inhibition (4 µM MYCi975, 72h). Data normalized to DMSO controls; mean ± SEM (n=3); unpaired t-test. **(C)** Volcano plot of differential dependencies between Complex I-low and -high cell lines. Negative Δ dependency scores indicate stronger dependency in Complex I-low cells. MYC (red) emerges as one of the most significant dependencies ($-\log_{10}$(P-value) vs Δ dependency score). Dotted lines mark p=0.05 and ΔDependency=±0.25. **(D)** Cell viability showing synthetic lethality between MYCi975 (4 µM) and inhibitors of pyruvate metabolism: R-GNE-140 (LDHA), CPI-613 (PDH), and PC-IN-1 (pyruvate carboxylase). LDHA inhibition produces strongest synergy. Mean ± SEM (n=3); one-way ANOVA with Tukey's test. **(E)** Correlation between MYC protein levels and Complex I gene expression in TCGA prostate cancer samples. Heatmap shows Spearman's correlation coefficients. **(F)** Kaplan-Meier analysis of progression-free survival in PRAD patients (n=493) stratified by Complex I expression. Patients with low Complex I expression (z-scores ≤2) show significantly better survival (log-rank test, p=3.235e-3).

FIGURE S3

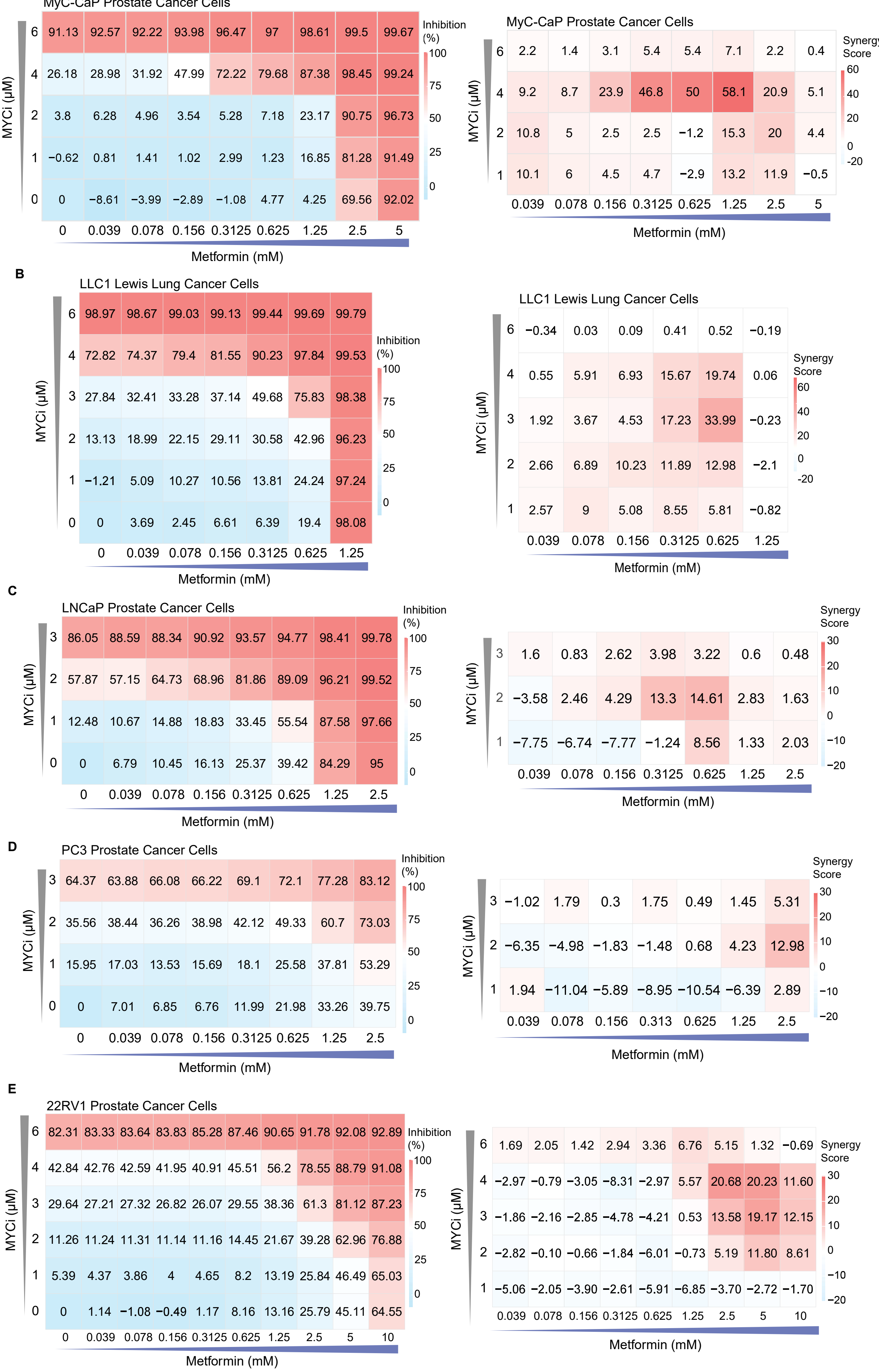

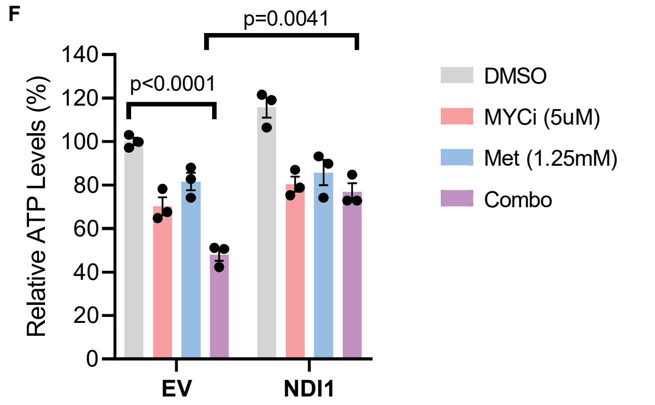

Figure S3. Synergistic interactions between MYCi975 and metformin across multiple cancer models.
(A to E) Synergy analysis of MYCi975 and metformin combinations in cancer cell lines. Left panels show growth inhibition (%) relative to vehicle; right panels show Bliss synergy scores. Data shown for MycCaP (A), LLC1 (B), LNCaP (C), PC3 (D), and 22RV1 (E) cells. Positive synergy scores (red) indicate greater-than-additive effects. Results represent mean of three independent experiments. **(F)** Cellular ATP levels in MycCaP cells expressing empty vector (EV) or NDI1 treated with DMSO, MYCi975 (5 μM), metformin (1.25 mM), or their combination for 24h. NDI1 expression specifically rescues ATP depletion in combination-treated cells. Data are normalized to DMSO control; mean ± SEM (n=3); unpaired t-test

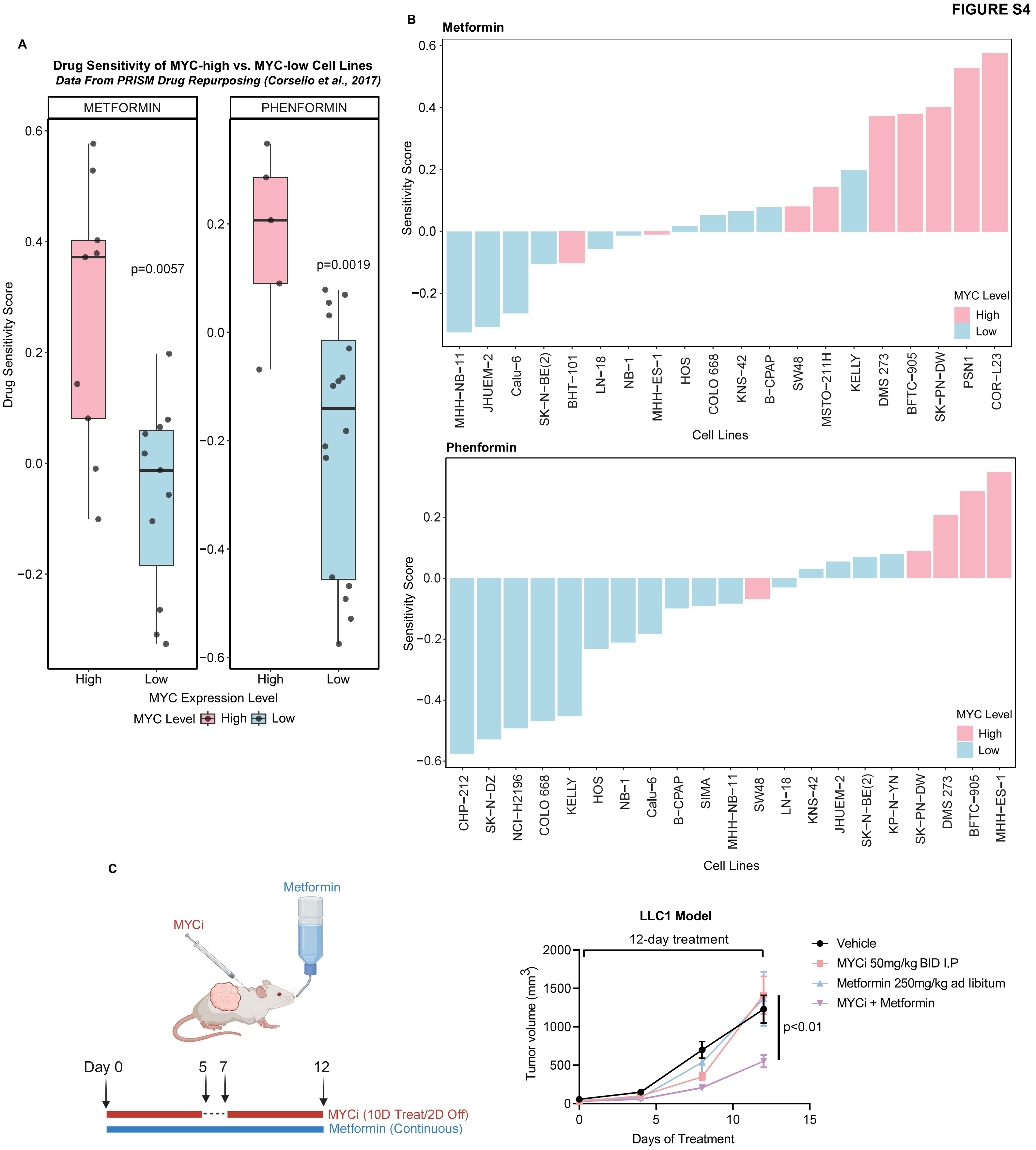

Figure S4. Validation of MYC-dependent metformin sensitivity. (**A)** Box plots from the PRISM Repurposing dataset showing drug-sensitivity scores for metformin and phenformin in MYC-high (top 5th percentile) versus MYC-low (bottom 5th percentile) cell lines (39). p=0.0057 for metformin and p=0.0019 for phenformin (two-tailed Student's t-tests). Boxes show medians and interquartile ranges; individual points represent distinct cell lines. Data adapted from the PRISM Repurposing Public dataset (39); graphic created by the authors. (**B)** Waterfall plots illustrating per-cell-line sensitivity to metformin (top) and phenformin (bottom). Cell lines are ordered by increasing drug--sensitivity score. Bars are colored by MYC expression category (blue, MYC-high; pink, MYC-low) (**C**) Left: Schematic of the in vivo LLC1 xenograft study, with MYCi975 (50 mg/kg, BID i.p.) on a 10-day schedule (2 days off) and continuous oral metformin (250 mg/kg/day). Right: Tumor growth curves over 12 days in LLC1 tumor-bearing mice (n=14/group). Data are mean ± SEM; p<0.01 by unpaired Student's t-test.

FIGURE S5

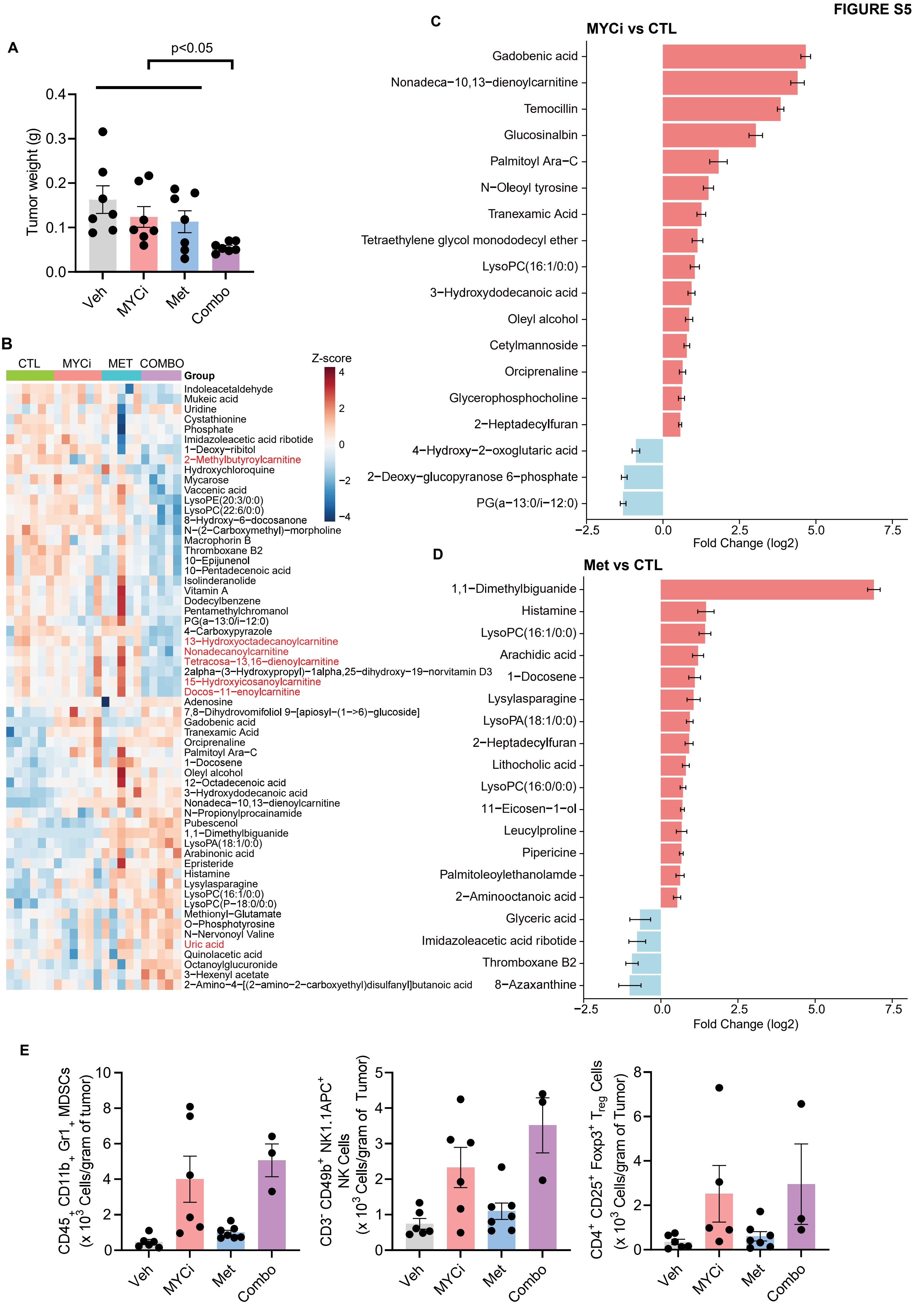

Figure S5. Distinct metabolomic signatures of MYCi975 and metformin treatments.
(A) Tumor weights from mice treated with vehicle, MYCi975, metformin or combination (n=7-8 per group). Data presented as mean ± SEM. $P < 0.05$ by one-way ANOVA with Tukey's post-hoc test. **(B)** Heatmap of untargeted metabolomics (z-score normalized) for all treatment groups. Notably, acyl-carnitine species (highlighted in red) are significantly reduced under combination therapy. **(C and D)** Differential metabolite abundance in MYCi975 versus vehicle (C) and metformin versus vehicle (D) treated tumors (log_2 fold change, n = 5-6 per group). **(E)** Flow cytometry quantification of $CD45^+$ $CD11b^+$ $Gr1^+$ MDSCs, $CD3^+$ $CD49b^+$ $NK1.1^+APC^+$ NK cells, and $CD4^+$ $CD25^+$ $Foxp3^+$ T regulatory cells in treated tumors (n=6-8 per group). Data presented as mean ± SEM. Statistical analysis by one-way ANOVA with Tukey's post-hoc test.

Supplementary Table Titles and Captions

Supplementary Table 1: Full Results of the MYCi975 CRISPR Synergy Screen in MycCaP Cells
Complete dataset from the genome-wide CRISPR knockout screen in MycCaP cells treated with MYCi975, including gene names, sgRNA sequences, normalized read counts, NormZ scores, and statistical parameters identifying synthetic lethal interactions.

Supplementary Table 2: Donut Plot Gene Annotations (Synergistic Hits, Mitochondrial Complex Components and MYC Targets)
Gene annotations used for the donut plot analysis in Figure 1D, categorizing mitochondrial respiratory chain complex components (I-V), MYC-regulated genes, and synergistic hits identified from the CRISPR screen.

Supplementary Table 3: DepMap Analysis of MYC Dependency in Complex I-Low vs. Complex I-High Cell Lines
Differential dependency analysis of MYC across cell lines stratified by Complex I expression levels from the DepMap AVANA dataset, including CERES scores, Δ dependency values, and statistical significance parameters referenced in Figure 2G.

Supplementary Table 4: RNA-Seq of MycCaP Cells Treated with MYCi975 (Mitochondrial Gene Clusters)
Differential gene expression data from RNA-sequencing of MycCaP cells treated with various concentrations of MYCi975, focusing on mitochondrial genes organized into the four clusters described in Figure 3J-K.

Supplementary Table 5: PRISM Drug-Sensitivity Scores for Metformin and Phenformin in MYC-High vs. MYC-Low Cell Lines
PRISM Repurposing dataset analysis comparing drug sensitivity scores for metformin and phenformin between MYC-high and MYC-low cell lines as shown in Figure S4A-B, including individual cell line data and statistical analysis.

Supplementary Table 6: Untargeted Metabolomic Profiles of MycCaP Tumors Treated with MYCi975, Metformin, or Both
Comprehensive metabolomic data from MycCaP tumors under different treatment conditions (vehicle, MYCi975, metformin, and combination), including metabolite identifications, abundance values, fold changes, and statistical analysis referenced in Figure 5A-B.

Supplementary Table 7: Cancer Atlas of Metabolic Profiles CAMP Metabolite–Immune Correlations in PRAD and Other Cancers
Correlation analysis between metabolites (particularly uric acid) and immune cell populations across multiple cancer types from the Cancer Atlas of Metabolic Profiles (CAMP) database, as referenced in Figure 5C-D.
